# Analysis of Biomarkers in Diabetic Foot Ulcer Patients With Dampness–Heat Syndrome Based on 4D‐DIA Proteomics Technology

**DOI:** 10.1155/jdr/6604989

**Published:** 2026-03-25

**Authors:** Jinlun Jiang, Shiyu Wang, Yiming Ni, Jiawei Feng, Mingmei Zhou, Cheng Zhao

**Affiliations:** ^1^ Shanghai Traditional Chinese Medicine Integrated Hospital, Shanghai University of Traditional Chinese Medicine, Shanghai, China, shutcm.edu.cn; ^2^ Institute of Interdisciplinary Integrative Medicine Research, Shanghai University of Traditional Chinese Medicine, Shanghai, China, shutcm.edu.cn

**Keywords:** 4D-DIA proteomics, biomarkers, cholesterol metabolism, dampness–heat syndrome, diabetic foot ulcer, traditional Chinese medicine

## Abstract

**Background:**

Diabetic foot ulcer (DFU), a severe complication of diabetes, impose substantial global health burdens. Dampness–heat syndrome (DHS), a common syndrome in traditional Chinese medicine (TCM), is highly prevalent among DFU patients and closely correlated with treatment response and prognosis. However, the molecular biomarkers associated with DFU in patients with DHS remain poorly understood.

**Methods:**

Serum 4D‐data‐independent acquisition (DIA) proteomics was performed on 16 DFU–DHS patients and six healthy controls (HCs). Differentially expressed proteins (DEPs) were screened by |fold change (FC)| > 1.2 and *p* < 0.05. Gene Ontology (GO), Kyoto Encyclopedia of Genes and Genomes (KEGG), and protein–protein interaction (PPI) analyses were conducted. Key biomarkers were validated via enzyme‐linked immunosorbent assay (ELISA) in 28 independent DFU–DHS cases.

**Results:**

A total of 201 DEPs were identified between DFU–DHS patients and HCs. Bioinformatics revealed DEPs enriched in lipid metabolism (high‐density lipoprotein [HDL] remodeling and cholesterol metabolism) and complement–coagulation cascades. PPI network analysis revealed a core functional module centered on four proteins, APOA1, LCAT, PLTP, and CETP. ELISA validation confirmed the significant dysregulation of these four apolipoproteins in the independent DFU–DHS cohort (all *p* < 0.05 vs. HCs). The combination of the biomarkers APOA1, LCAT, PLTP, and CETP exhibited a high diagnostic efficacy for DFU–DHS, with an area under the curve (AUC) of 0.9672 based on receiver operating characteristic (ROC) analysis.

**Conclusion:**

To our knowledge, this is the first study to employ 4D‐DIA proteomics on DFU–DHS. We identified four serum biomarkers (APOA1, LCAT, PLTP, and CETP) linked to dysregulated cholesterol metabolism in DFU–DHS patients, which show diagnostic potential and provide insights for integrating TCM syndrome differentiation with precision medicine.

## 1. Introduction

Diabetic foot ulcer (DFU), a severe complication of diabetes mellitus, is associated with substantial morbidity, mortality, and a heavy global healthcare burden [1, 2]. Globally, the annual incidence of DF ranges from 1.0%−4.1%, with a lifetime risk of 19%–34%, and approximately 15% of patients with diabetes develop DFU [3]. Pathophysiologically, DFU arises from the triad of peripheral neuropathy, ischemia, and infection. Nearly 50% of DFUs progress to infection, and 20% of patients with moderate‐to‐severe infection require lower‐limb amputation. The 5‐year mortality rate postamputation remains as high as 50%–70% [4]. DFU also imposes a considerable economic burden, with global annual costs exceeding 10.9 billion. In China, the cost of DFU treatment is projected to increase from the current $4.9 billion to $7.4 billion by 2030 [5–7], driven by prolonged hospitalization, surgical interventions, and rehabilitation [8, 9]. Despite advances in standard clinical care, strategies for the early diagnosis and effective treatment of DFU remain inadequate, highlighting the urgent need to unravel novel mechanistic insights and develop improved therapeutic approaches.

Traditional Chinese medicine (TCM), characterized by its holistic perspective and multitarget interventions, provides a complementary approach to DFU management [10]. The core of TCM practice is “Syndrome differentiation (Bian Zheng),” which classifies patients on the basis of distinct pathophysiological manifestations. Dampness–heat syndrome (DHS) is recognized as a pivotal syndrome type in DFU pathogenesis. Epidemiological analysis has confirmed that there is a significant correlation between dampness–heat constitution (9.08% of the Chinese population) and an increased risk of diabetes [11]. In type 2 diabetes (T2D) mellitus, “Dampness–heat trapping the spleen” is the most common syndrome (58.29%), followed by “Qi‐Yin deficiency” (16.03%) and “Yin deficiency with heat” (12.93%) [12]. The Guidelines for the Diagnosis and Treatment of Diabetic Foot Disease Based on TCM (2021) highlight DHS as a core syndrome of DFU, recommending integrated treatment protocols that combine TCM heat‐clearing and dampness–resolving therapies with conventional Western medicine [13–15]. The core pathogenesis of DFU–DHS involves “Spleen deficiency,” which impairs fluid metabolism and transport, leading to internal dampness accumulation, which may further transform into heat over time. Clinically, DFU–DHS is characterized by local redness, swelling, heat, pain, and exudation in the foot and is hypothesized to drive disease progression through promoting chronic inflammation, metabolic dysfunction, and impaired immunity. Thus, elucidating biomarkers for the precise stratification of DFU patients with DHS stratification is critical for identifying DFU pathogenesis and optimizing intervention strategies.

However, the molecular mechanisms underlying DFU–DHS, particularly its systemic proteomic characteristics, remain largely unexplored, resulting in a significant knowledge gap in understanding this DFU subtype. High‐throughput proteomics provides a powerful tool to address this gap. Data‐independent acquisition (DIA) mass spectrometry (MS) exhibits notable advantages in biomarker discovery, including comprehensive protein coverage, high quantitative accuracy, and excellent reproducibility [16]. Notably, the recent integration of ion mobility separation, termed as four‐dimensional DIA (4D‐DIA) proteomics, has been demonstrated to improve sensitivity and specificity by introducing a fourth dimension of separation, significantly enhancing data quality [17]. In the present study, we employed 4D‐DIA proteomics to characterize the serum protein expression profile of DFU patients with DHS, investigated the biological significance of protein expression alterations in this subtype, and subsequently performed bioinformatics analysis and enzyme‐linked immunosorbent assay (ELISA) validation of candidate protein biomarkers.

## 2. Materials and Methods

### 2.1. Study Design and Participant Recruitment

This case–control study was conducted between October 2021 and November 2023 at Shanghai TCM Integrated Hospital. The study’s sample population included 16 participants with DFU who presented with DHS and 6 healthy control (HC) subjects. The study protocol was approved by the Institutional Review Board of Shanghai Combined Traditional Chinese and Western Medicine Hospital (Approval Number 2021‐083‐1), and all participants provided written informed consent prior to enrollment.

The diagnosis of DFU was established according to the World Health Organization guidelines. TCM syndrome differentiation was performed by two certified TCM practitioners based on the criteria outlined in the Clinical Research Guiding Principles for the Treatment of Diabetes with TCM New Drugs (2002 edition). The key diagnostic criteria for DHS included: heavy sensation in the head and body, epigastric fullness, thirst with desire to drink little, a plump and tender tongue with a yellow‐greasy coating, and a slippery or rapid pulse. The detailed inclusion and exclusion criteria for DFU, DFU–DHS and HC groups are summarized in Table 1.

**Table 1:** DFU–DHS inclusion and exclusion criteria.

DFU criteria: For the patients who met the requirements, two trained professionals collected basic information and TCM symptoms for TCM syndrome differentiation and syndrome analysis using the basic information collection table of type 2 diabetes and information collection table of four diagnostic methods of TCM symptoms. Syndrome type classification and diagnosis were based on the investigation results of an expert group, who used the State Administration of Traditional Chinese Medicine Clinical Research Guiding Principles for the Treatment of Diabetes with Traditional Chinese Medicine New Drugs (2002 edition).
DFU–DHS Inclusion criteria: (1) Age: 16–80 years. (2) Diagnosed with type 2 diabetes mellitus and DFU (Wagner grade 3 or 4). (3) Recurrent DFU (≥ 2 episodes). (4) Ankle‐brachial index (ABI) < 0.6. (5) Met the TCM diagnostic criteria for dampness–heat syndrome (DHS), characterized by at least three of the following: heavy sensation in the head/body, epigastric fullness, thirst with little desire to drink a plump and tender tongue with yellow‐greasy coating, and a slippery or rapid pulse. (6) Informed consent of the patient had been obtained and able to adhere to treatment and follow‐up as scheduled.
Exclusion criteria: (1) Comorbidity with diseases affecting the healing of DFU, such as a tumor, osteomyelitis, vasculitis, immune system disorder, et cetera, or diagnosis of severe liver dysfunction, end‐stage renal disease, long‐term treatment with radiotherapy, chemotherapy, and growth factor drugs, poor blood supply conditions. (2) Foot ulcers caused by other causes other than diabetes. (3) Study participants with DFU who did not provide informed consent. (4) Study participants with severe cardiopulmonary and renal complications. (5) Study participants with severe hypertension (hypertension crisis or malignant hypertension), cardiovascular and cerebrovascular diseases (such as cardiac function grade IV, sequelae of severe cerebrovascular diseases), and patients diagnosed with acute infection and infectious diseases. (6) Other limb ischemic diseases, such as lower limb vascular stenting, amputation, vascular obliteration vasculitis, Takayasu arteritis, Raynaud’s disease, and cold injured vascular disease.
HC
Inclusion criteria: (1) Age: 16–80 years. (2) Foot wounds, not caused by diseases. (3) Absence of chronic diseases or surgery.
Exclusion criteria: (1) Medication within 1 month before participation in the study. (2) Study participants taking any surgery. (3) Diagnosis of other diseases, such as diabetic foot, hypertension, and nephropathy.

Abbreviations: DFU, diabetic foot ulcer; DHS, dampness–heat syndrome; HC, healthy control; TCM, traditional Chinese medicine.

### 2.2. Serum Sample Collection and Preparation

Peripheral blood (5 mL) was obtained from each participant following an overnight fasting period. The blood samples were collected in EDTA‐containing tubes and subjected to processing within 2 h after collection. Centrifugation was performed at 4°C and 3500 rpm for 10 min to separate the serum fraction. The resulting serum was divided into 200 *μ*L aliquots in sterile Eppendorf tubes and promptly stored at −80°C to avoid multiple freezing and thawing events.

**Figure 1:**
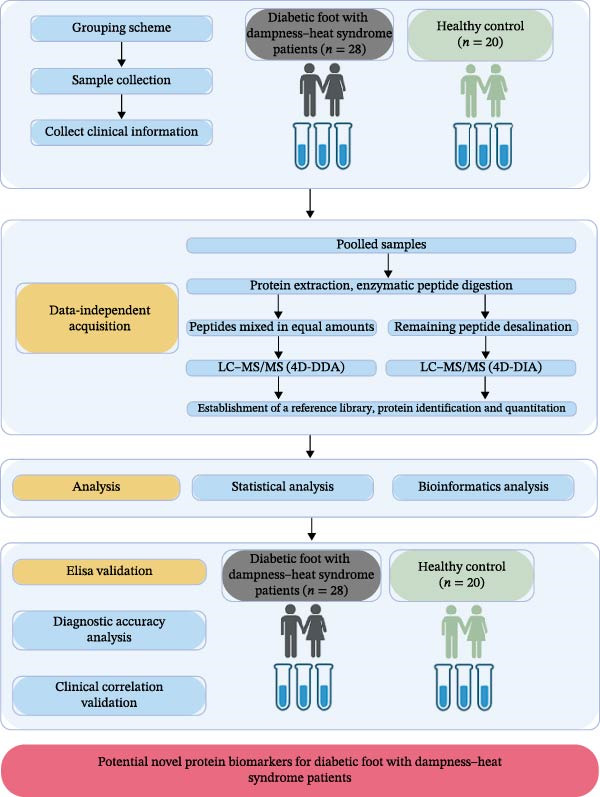
The flow diagram of analysis. The abstract diagram describes the study design process, including grouping information, experimental flow, and how to identify potential protein biomarkers of diabetic foot ulcer patients with dampness–heat syndrome patients.

Prior to analysis, frozen serum samples were thawed a single time to preserve protein integrity. Total protein was extracted from the processed serum, and a portion was reserved for determining protein concentration and assessing quality via SDS–PAGE. The remaining sample underwent tryptic digestion. Subsequent to this, the digested peptides underwent a process of desalting and were then subjected to analysis through the utilization of a liquid chromatography–tandem MS (LC–MS/MS) approach. The LC–MS/MS workflow comprised two key phases: first, a protein spectral library was constructed using data‐dependent acquisition (DDA), and second, MS data for individual samples were acquired via DIA. Quantitative data extraction and spectral matching were then conducted by referencing the DDA‐derived spectral library, followed by statistical analysis. The experimental workflow is schematically represented in Figure 1.

### 2.3. Protein Extraction and Peptide Enzymolysis

The aim is to enhance the detection of low‐abundance proteins, serum proteins were extracted and purified using a commercial albumin/IgG depletion kit (Melon Gel, Thermo Scientific) according to the manufacturer’s instructions. Briefly, 30–60 *μ*L of serum was diluted with 270–540 *μ*L of provided binding buffer. The mixture was applied to a spin column and passed through the resin bed by gravity flow. After washing with binding buffer, the flow‐through containing the depleted serum was collected. A bicinchoninic acid assay kit (Pierce)was used to determine the total protein concentration of the depleted serum. A standard serum albumin standard curve was generated, and the absorbances were measured at 562 nanometers using a microplate reader. To ascertain protein quality and integrity, an SDS–PAGE method with 12% acrylamide was implemented, followed by a Coomassie blue staining procedure.

### 2.4. Proteolytic Digestion and Peptide Desalting

In the experiment, 100 *μ*g of protein from each sample underwent reduction with 5 mM dithiothreitol (56°C, 30 min) and alkylated in the dark with 10 mM iodoacetamide (room temperature, 15 min). Proteins were then precipitated via overnight incubation with six volumes of pre‐chilled acetone at −20°C. The resulting pellets were subsequently collected via a centrifugation process (8000 × *g*, 10 min, 4°C) and thoroughly redissolved in 50 mM ammonium bicarbonate. Trypsin from Promega was added at a ratio of 1:50 (w/w) of enzyme to substrate. The digestion was conducted at a temperature of 37°C for a period of 12 h.

Following digestion, the peptides were purified using SOLA SPE 96‐well plates (Thermo Scientific). The plates were activated with methanol and equilibrated with water prior to sample loading. Peptides underwent extraction with an aqueous solution comprising 80% acetonitrile and 0.1% formic acid. Following the collection of the extracted peptides, the eluate underwent a drying process under vacuum. The lyophilized peptides were resuspended in 0.1% formic acid. This mixture was used for subsequent LC–MS/MS analysis.

### 2.5. 4D‐DIA MS and Data Acquisition

LC–MS/MS analyses were conducted on a timsTOF mass spectrometer (Bruker Daltonics) coupled to a nanoElute UHPLC system (Bruker). Peptide separation was carried out using a reversed‐phase C18 column (IonOpticks, 25 cm × 75 *μ*m, 1.6 *μ*m) with a 60 min linear gradient delivered at 300 nL/min. The mobile phase consisted of a solution of 0.1% formic acid in water (component A) and a solution of 0.1% formic acid in acetonitrile (component B). The gradient profile was as follows: the initial ratio was set at 2%–22% B over 45 min, followed by an increase to 22%–35% B over 10 min. The ratio was then increased to 80% B for 5 min and maintained at that level for an additional 5 min.

The acquisition of MS data was executed through the implementation of a DIA parallel accumulation–serial fragmentation mode. The ion mobility range was established from 0.7 to 1.3 Vs/cm^2^, and the MS scans encompassed a m/z range of 100–1700. The collision energy was adjusted in a linear fashion, contingent upon the ion mobility.

### 2.6. Database Search and Protein Quantification

The raw DIA files were processed in Spectronaut Pulsar (v15.3) using the human UniProt database. The search parameters included tryptic peptides as the protease. These could have up to two missed cleavages. Carbamidomethylation of cysteine was also included as a fixed modification. Oxidation of methionine and N‐terminal acetylation were included as variable modifications. The control of false discovery rates at both the peptide and protein levels was achieved with a limit of ≤1%.

Label‐free quantification was performed using the software’s integrated algorithm. Proteins with missing values in over 50% of the samples in either the DFU–DHS or HC group were removed. The data were normalized by median centering and log_2_‐transformed prior to statistical analysis.

### 2.7. Bioinformatic and Statistical Analysis

DEPs between the DFU–DHS and HC groups were defined as those exhibiting |log_2_ fold change (FC)| ≥ 0.263 and an adjusted *p*‐value < 0.05 (Student’s *t*‐test with Benjamini–Hochberg correction). Functional enrichment analysis for Gene Ontology GO (https://www.blast2go.com/) terms and Kyoto Encyclopedia of Genes and Genomes (KEGG; https://www.genome.jp/kegg/pathway.html) pathways was conducted using the clusterProfiler package in R. A protein–protein interaction (PPI) network was generated using the STRING database (v11.5, confidence score > 0.7) and visualized in Cytoscape (v3.10.3). Key functional modules within the network were identified using the MCODE plugin.

### 2.8. ELISA Validation

An independent validation cohort comprising 28 DFU–DHS patients was recruited. The serum levels of four candidate biomarker proteins (APOA1, LCAT, CETP, and PLTP) were quantified using available ELISA kits (Shanghai KCW Tech Co., Ltd., China; Cat. No. LCAT: ARD2146; APOA1: ARD11500; PLTP: KCW03‐105; CETP: KCW19845) strictly following the manufacturers’ protocols.

### 2.9. Statistical Analysis for Validation

All statistical analyses for the validation cohort were conducted using the software program SPSS (version 26.0). Continuous data are summarized as mean ± standard error of the mean (SEM). The diagnostic efficacy of individual biomarkers and their combinations was appraised through the generation of receiver operating characteristic (ROC) curves. The area under the curve (AUC) was employed as the metric of performance. Associations between protein expression levels and clinical parameters were examined using Pearson correlation analysis. A two‐sided *p*‐value of less than 0.05 was defined as statistically significant.

## 3. Results

### 3.1. Overview of Proteomic Profiling

The discovery cohort included 16 DFU patients (eight women and eight men), 16 DFU–DHS patients (eight women and eight men) and six HCs (three women and three men). There was no significant difference in age or gender distribution between the DFU–DHS group and the HC group (*p* > 0.05). As expected, the DFU–DHS patients presented significantly elevated inflammatory and metabolic markers relative to controls. Furthermore, comparative analysis reveals that DHS patients exhibit higher levels of acute‐phase reactants (C‐reactive protein [CRP] and ESR) and a more severe depression of high‐density lipoprotein cholesterol (HDL‐C) than diabetic foot patients without DHS symptoms. The full baseline clinical characteristics are provided in Table 2.

**Table 2:** Baseline characteristics of DFU–DHS group and HC group.

Variable	Reference range	Discovery			DFU–DHS vs. HC *p*‐value	Validation		DFU–DHS vs. HC *p*‐value
		DFU	DFU–DHS	HC		DFU–DHS	HC	
		*n* = 16	*n* = 16	*n* = 8		*n* = 28	*n* = 20	
Age (in year) ^∗^	—	63.26 ± 5.30	65.50 ± 8.07	66.00 ± 4.87	0.85	61.89 ± 6.92	64.25 ± 6.41	0.23
Male	—	8 (50%)	8 (50%)	3 (50%)	1	14 (50%)	10 (50%)	1
Female	—	8 (50%)	8 (50%)	3 (50%)	1	14 (50%)	10 (50%)	1
CRP (mg/dL)	< 0.5	42.68 ± 35.46	64.44 ± 35.46	3.38 ± 0.99	≤ 0.001 ^∗^	68.53 ± 41.41	1.03 ± 1.76	≤ 0.001 ^∗^
WBC (× 10^9^/L)	4.0–10.0	12.94 ± 4.93	11.27 ± 5.09	6.98 ± 1.33	0.005 ^∗^	11.58 ± 5.17	6.57 ± 1.47	≤ 0.001 ^∗^
ESR (mm/H)	<15	48.63 ± 23.37	62.94 ± 41.31	7.38 ± 2.45	≤ 0.001 ^∗^	61.68 ± 35.91	6.83 ± 2.42	≤ 0.001 ^∗^
TC (mmol/L)	<5.18	7.38 ± 2.77	6.49 ± 1.93	3.47 ± 1.04	0.011 ^∗^	6.43 ± 2.17	2.84 ± 1.20	≤ 0.001 ^∗^
TG (mmol/L)	<1.7	1.64 ± 1.78	1.59 ± 1.04	1.22 ± 0.29	0.219	1.39 ± 0.61	1.48 ± 0.33	0.525
HDL‐C	0.8–1.8	0.85 ± 0.25	0.76 ± 0.33	1.40 ± 0.26	≤ 0.001 ^∗^	0.57 ± 0.17	1.54 ± 0.49	≤ 0.001 ^∗^
LDL‐C (mmol/L)	< 3.37	4.02 ± 1.41	3.58 ± 1.51	2.08 ± 0.53	0.002 ^∗^	3.86 ± 1.87	2.12 ± 0.51	≤ 0.001 ^∗^
VLDL‐C (mmol/L)	0.24–1.39	0.93 ± 0.87	0.88 ± 0.63	0.63 ± 0.27	0.189	0.51 ± 0.26	0.49 ± 0.14	0.72
HbA1c (%)	3.6–6.0	10.03 ± 4.48	9.51 ± 3.03	4.58 ± 0.79	≤ 0.001 ^∗^	9.52 ± 2.74	4.52 ± 0.92	≤ 0.001 ^∗^
FBG (V)	3.9–6.1	6.26 ± 5.37	6.45 ± 4.68	4.95 ± 0.52	≤ 0.001 ^∗^	5.98 ± 2.02	4.97 ± 0.62	0.018 ^∗^
2hBG (V)	3.6–7.8	19.31 ± 5.42	16.00 ± 6.79	6.37 ± 0.77	≤ 0.001 ^∗^	14.03 ± 6.65	7.06 ± 2.45	≤ 0.001 ^∗^

*Note*: Data were expressed as mean ± SEM,  ^∗^ *p* < 0.05 vs. HC. 2hBG, 2 h postprandial blood glucose; HbA1c, glycated hemoglobin.

Abbreviations: CRP, C‐reactive protein; ESR, erythrocyte sedimentation rate; FBG, fasting blood glucose; LDL, low‐density lipoprotein; TC, total cholesterol; TG, triglycerides; VLDL, very low‐density lipoprotein; WBC, white blood cells.

To systematically investigate the proteomic alterations associated with DFU in patients with DHS, we performed a quantitative proteomic analysis of 22 serum samples (from 16 DFU–DHS patients and six HCs) using DIA‐MS. Figure 1 illustrates the experimental and computational workflow for the 4D‐DIA‐based proteomic profiling.

### 3.2. Differentially Expressed Proteins (DEPs) Between Diabetic Foot Patients With DHS and HCs

The DIA proteomic dataset identified a total of 761 proteins. Principal component analysis (PCA) revealed a clear distinction between the DFU–DHS and control group, with the first principal component explaining 22.7% of the total variance. These findings suggest a distinct proteomic signature between the two groups (Figure 2A). Volcano plots were generated based on the criteria of |log_2_ (FC)| ≥ 0.263 and *p*‐value < 0.05 (Figure 2B). In total, 201 DEPs, including 53 upregulated DEPs and 148 downregulated DEPs were shown in Table S1. For example, serum amyloid A1 (SAA1), SAA2, CRP, fibrinogen like 1 (FGL1), and S100 calcium binding protein A8 (S100A8) were significantly upregulated, but multivesicular body subunit 12B (MVB12B) was downregulated in DFU compared to HC, suggesting that these proteins might be markers that can distinguish the diabetic feet with DHS group from those in HC group. As major acute‐phase reactants, SAA1/SAA2 levels increase up to 1000‐fold during inflammation [18], and are implicated in chronic inflammatory diseases, including atherosclerosis and rheumatoid arthritis [19]. Similarly, in type 1 diabetes (T1D), SAA‐associated inflammation in HDLs is exacerbated by poor glycemic control, with HDL3‐SAA increasing by 23% per 1% increase in HbA1c [20]. Specifically, elevated CRP is a well‐established biomarker of systemic inflammation in T2D and cardiovascular diseases [21]. However, CRP itself is not directly therapeutic; its reduction reflects attenuated inflammatory pathways [22]. S100A8 is elevated in individuals with T2D and obesity, where it exacerbates inflammation through activation of the TLR4/RAGE signaling pathway [23], contributing to *β*‐cell impairment, insulin resistance, and the development of complications such as diabetic nephropathy and retinopathy. In the context of nonalcoholic fatty liver and its inflammatory stage, nonalcoholic steatohepatitis, the significantly upregulated FGL1 activates the ERK1/2 signaling pathway, thereby leading to insulin resistance and liver, contributing to the systemic metabolic burden in DFU–DHS patients [24]. These significant changes in the mediators indicate that in patients with DFUs, the DHS exhibits distinct characteristics of systemic inflammation and lipid disorders.

Figure 2Overview of proteomic profiling. (A) PCA map of DEPs for all samples in diabetic foot ulcer with dampness–hot syndrome and healthy control group. (B) The volcano plot visualizes the protein distribution based on the fold change and the *p*‐value in diabetic foot. There are 58 upregulated differential proteins and 142 downregulated differential proteins, and dashed lines indicate the threshold values; the horizontal line marks a *p*‐value <0.05, while the vertical lines mark |log_2_ FC| > 0.263. (C–H) Boxplot showing the protein expression level between SZ and HC of top six upregulated or downregulated protein (|log_2_ FC| > 2, including SAA2, SAA1, CRP, FGL1, S100A8, and MVB12B.  ^∗^ *p* < 0.05;  ^∗∗^ *p* < 0.01;  ^∗∗∗^ *p* < 0.001.

**Figure:**
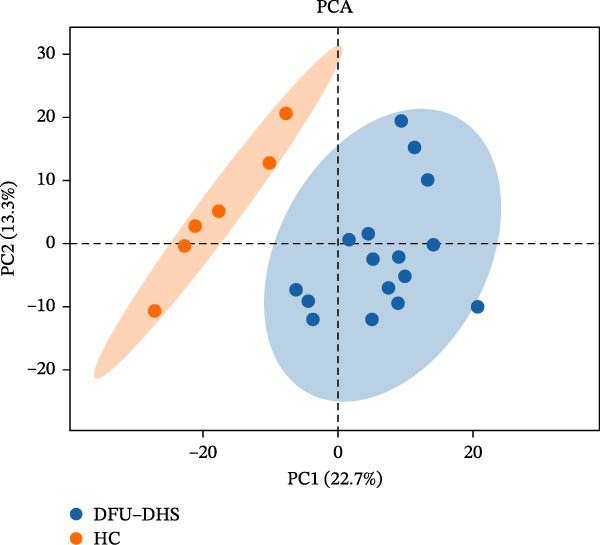
(A)

**Figure:**
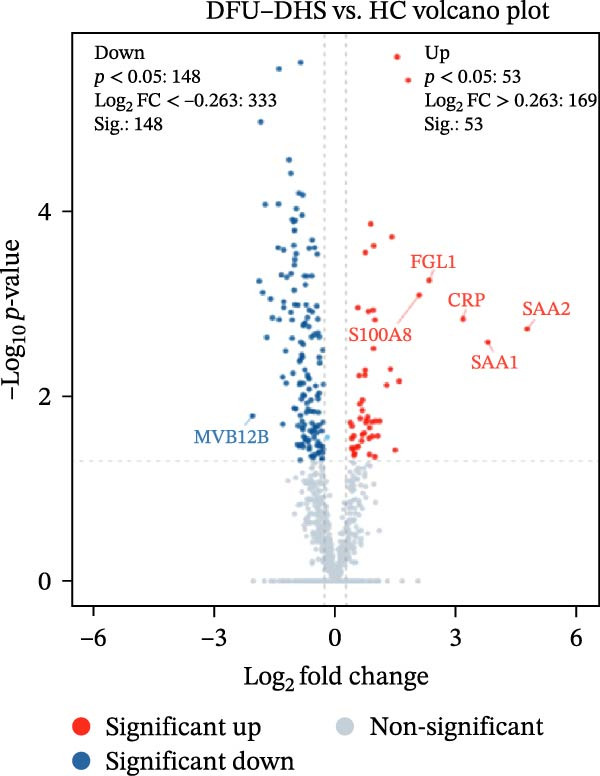
(B)

**Figure:**
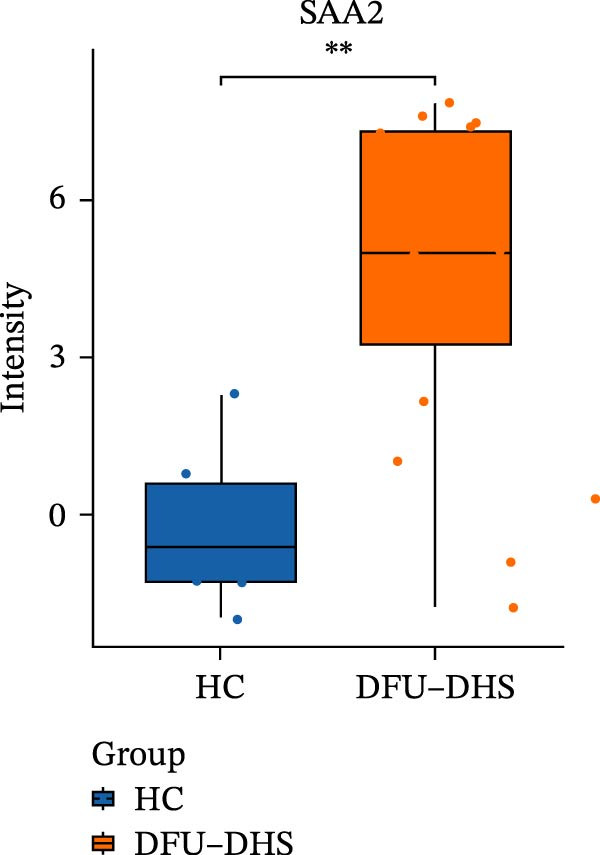
(C)

**Figure:**
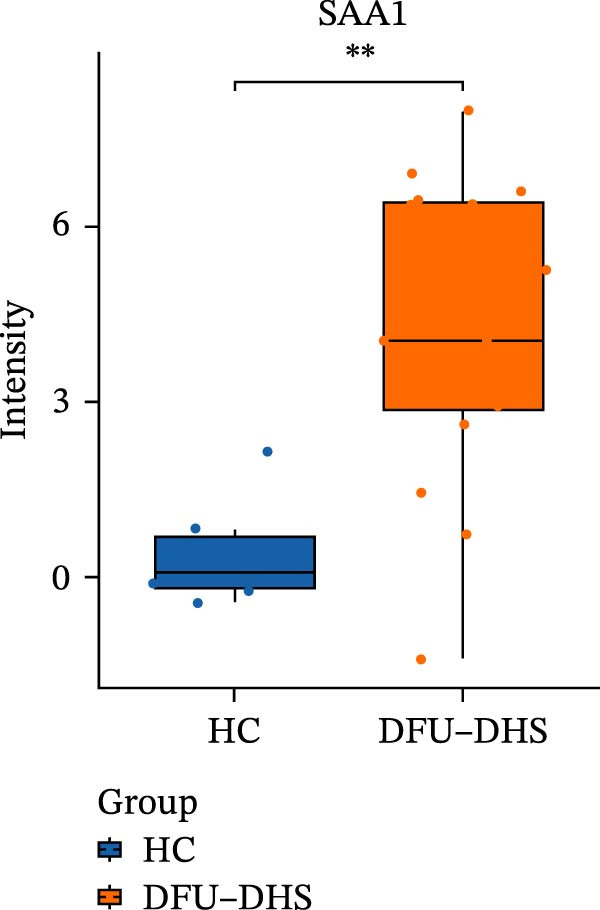
(D)

**Figure:**
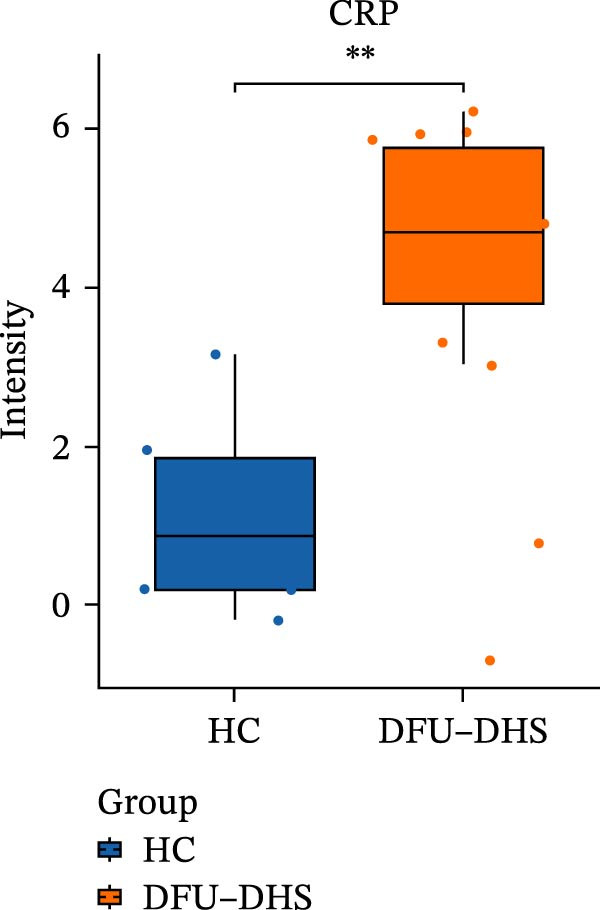
(E)

**Figure:**
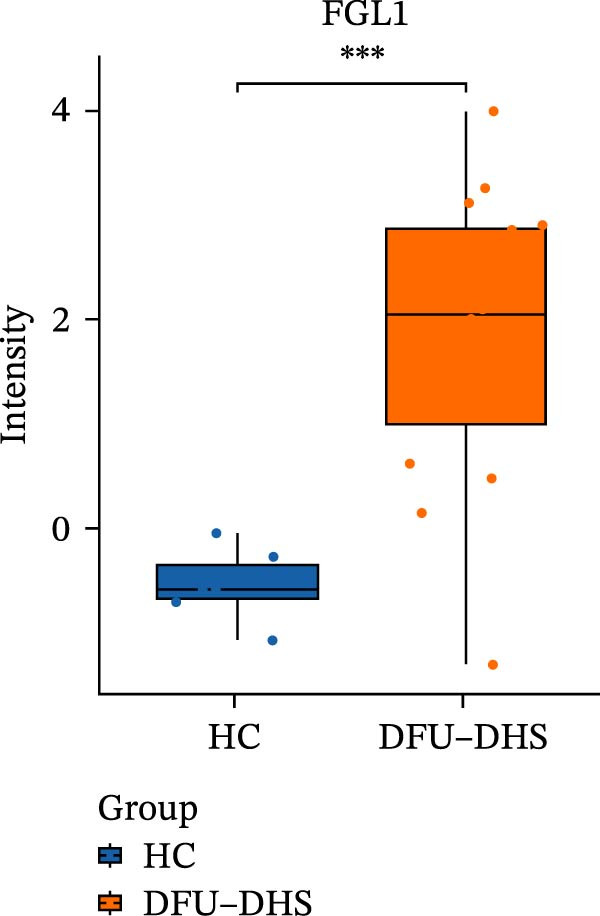
(F)

**Figure:**
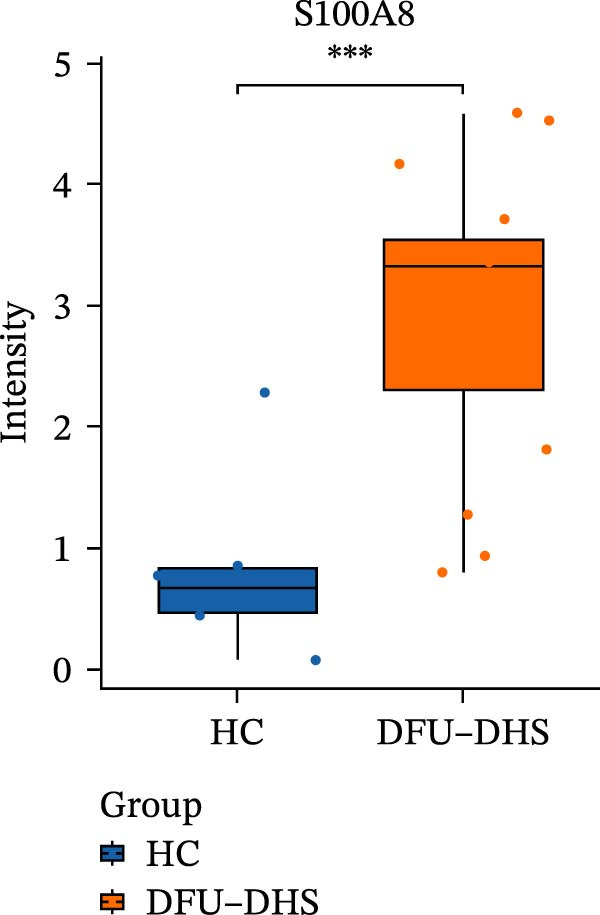
(G)

**Figure:**
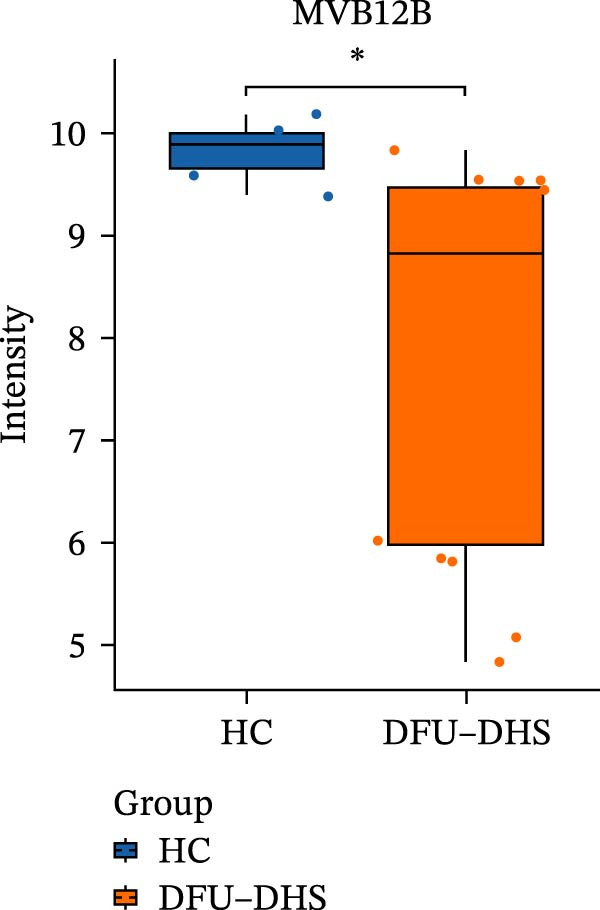
(H)

### 3.3. Functional Enrichment Analyses of DEPs

To explore the biological roles of DEPs, GO analysis was conducted to assess the functional implications of these proteins in patients with diabetic foot complicated by DHS compared with healthy individuals. The top 10 enriched GO terms in the biological process (BP), cellular component (CC), and molecular function (MF) categories are presented in Figure 3A. Within the BP category, the majority of DEPs were primarily associated with HDL particle remodeling, reverse cholesterol transport, and the acute‐phase response. The most significantly enriched CC terms included “blood microparticle”, “collagen‐containing extracellular matrix”, “exosome”, and other related compartments. In MF, the top terms were serine‐type endopeptidase activity, calcium ion binding, and heparin binding. To further evaluate the significant signaling associated with DEPs between the DFU–DHS and HC groups, KEGG pathway analysis was performed. The top enriched KEGG pathways between the DFU–DHS and HC group are illustrated in Figure 3B, including the complement and coagulation cascades, cholesterol metabolism, ECM–receptor interaction and cell adhesion molecules

Figure 3GO and KEGG analysis. (A) Top 10 GO terms (biological process, cellular component, and molecular function) associated with DEPs. Top 10 significant GO terms in biological process are shown in red, while those in cellular component in green and molecular function in blue. (B) Scatter plot for top 20 pathways in KEGG enrichment of DEPs. The enrichment score was calculated according to the number of annotated genes and that of all annotated genes in this pathway term, see method in detail. Lower *p*‐values indicate higher pathway enrichment. (C) Heat maps showing expression of DEPs related with high‐density lipoprotein in particle remodeling and cholesterol metabolism.

**Figure:**
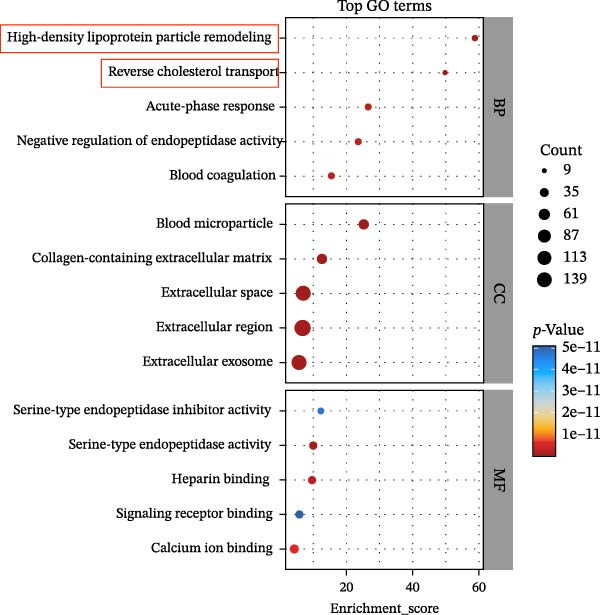
(A)

**Figure:**
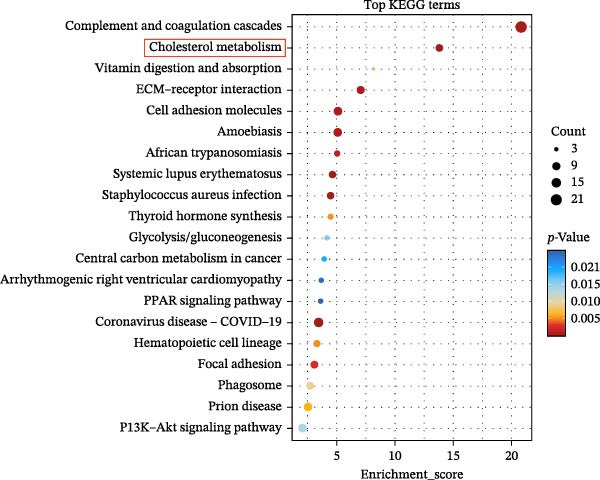
(B)

**Figure:**
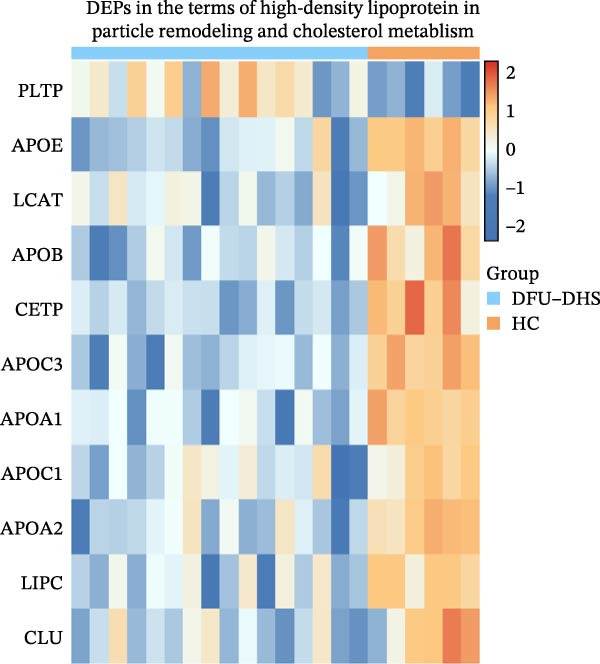
(C)

The heatmap plot of HDL particle remodeling and cholesterol metabolism revealed that most HDL particle remodeling related genes, including APOA1, LCAT, CETP, and PLTP were significantly dysregulated in the DFU–DHS group (Figure 3C). These results indicate that HDL particle remodeling and cholesterol metabolism are closely associated with DHS.

### 3.4. Research on the Metabolic Pathways of DEPs

KEGG analysis showed that the DEPs were enriched mainly in pathways of lipid metabolism, complement and coagulation, vitamin digestion and absorption, ECM–receptor interaction, and cell adhesion molecules (Figure 3B). Among them, the involvement of HDL particle remodeling and cholesterol metabolism drew our attention. Diabetic DHC is centered on spleen deficiency and dampness–heat internalization. The spleen is the master of transportation and digestion, and dampness–heat encumbering the spleen leads to impaired lipid metabolism, manifested by increased triglyceride (TG) synthesis and decreased lipoprotein clearance, which is closely related to the generation and accumulation of very low‐density lipoprotein (VLDL) and the celiac remnant (CM remnant). Moreover, patients with DHS often exhibit insulin resistance, leading to a reduction of HDL, increased hepatic VLDL secretion and decreased TG uptake by peripheral tissues. As shown in Figure 4A, the downregulated APOA1 is the predominant apolipoprotein in HDL. Additionally, CETP and LCAT are transfer protein between VLDL, IDL, LDL, and HDL in metabolic pathways, which are significantly change in DFU–DHS (Figure 4B–E).

Figure 4Disrupted HDL metabolism and its pro‐inflammatory shift in diabetic foot ulcer with dampness–heat syndrome (DFU–DHS). (A) Schematic diagram contrasting normal HDL metabolism with the dysfunctional state in DFU–DHS. Normal State (left): mature HDL particles, formed through a process involving APOA1, LCAT, and lipid transfer proteins (CETP and PLTP), facilitate reverse cholesterol transport (RCT) from macrophages (e.g., via ABCA1 and SR‐B1). This process exhibits anti‐inflammatory effects, including suppression of pro‐inflammatory cytokine production (e.g., IL‐6 and TNF‐*α*) via pathways like NF‐*κ*B. DFU–DHS state (right): in DFU–DHS, key proteins in the RCT pathway (APOA1, LCAT, CETP, and PLTP) are downregulated. This impairment is exacerbated by an acute‐phase response, where elevated serum amyloid A (SAA) binds to HDL, displacing APOA1 and further impairing its function. The resulting dysfunctional HDL loses its anti‐inflammatory and cholesterol efflux capacities, contributing to chronic inflammation and dyslipidemia in the wound microenvironment. (B–E) Box plots validate the significant downregulation of core HDL‐metabolism proteins. (B) APOA1, (C) LCAT, (D) PLTP, and (E) CETP in the DFU–DHS group (SZ, *n* = 16) compared to the healthy control group (HC, *n* = 6). These quantitative proteomic data underpin the pathological model depicted in (A).  ^∗^ *p* < 0.05;  ^∗∗^ *p* < 0.01;  ^∗∗∗^ *p* < 0.001.

**Figure:**
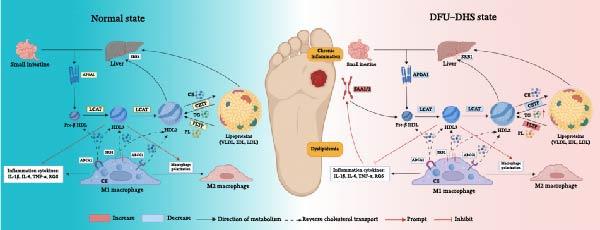
(A)

**Figure:**
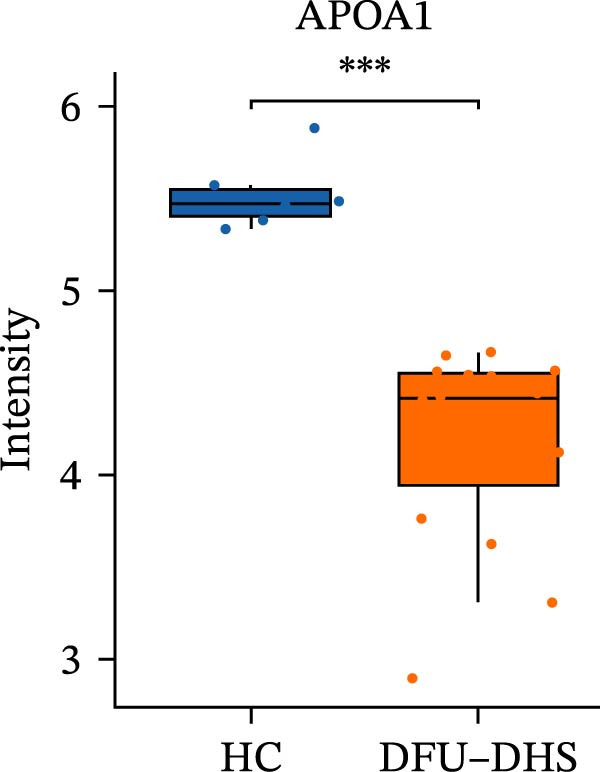
(B)

**Figure:**
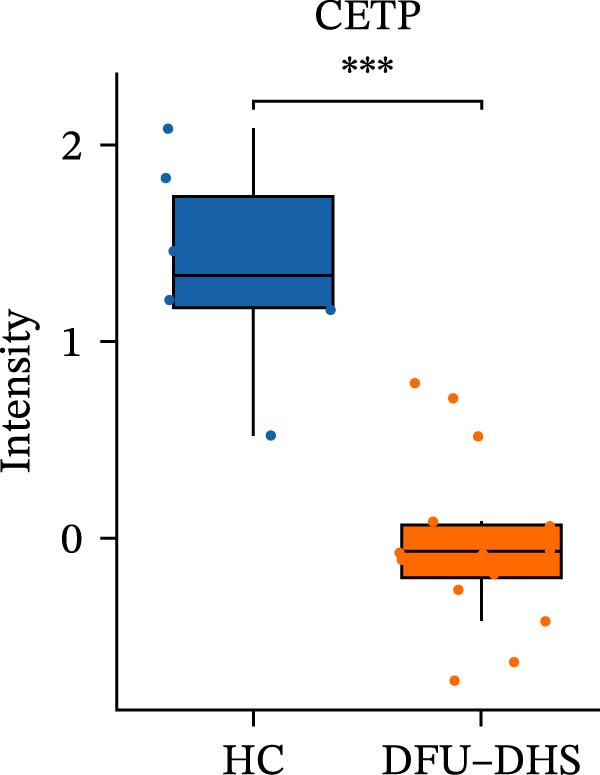
(C)

**Figure:**
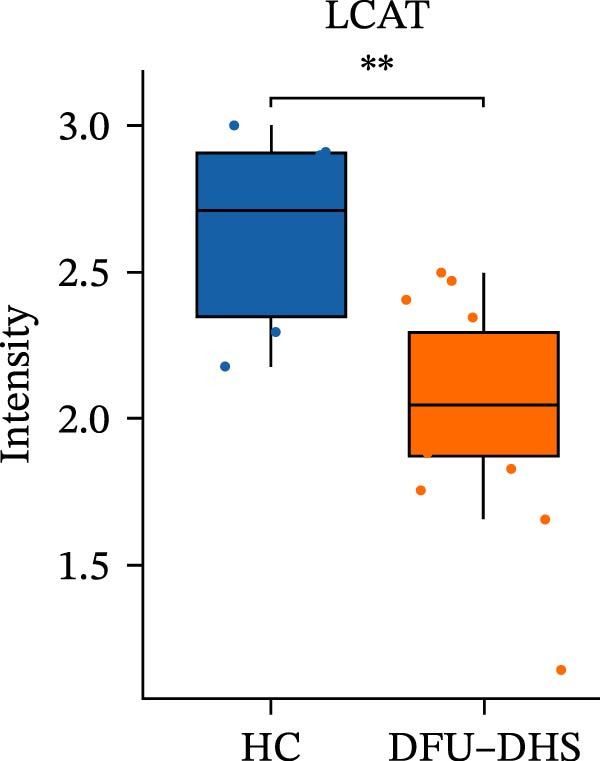
(D)

**Figure:**
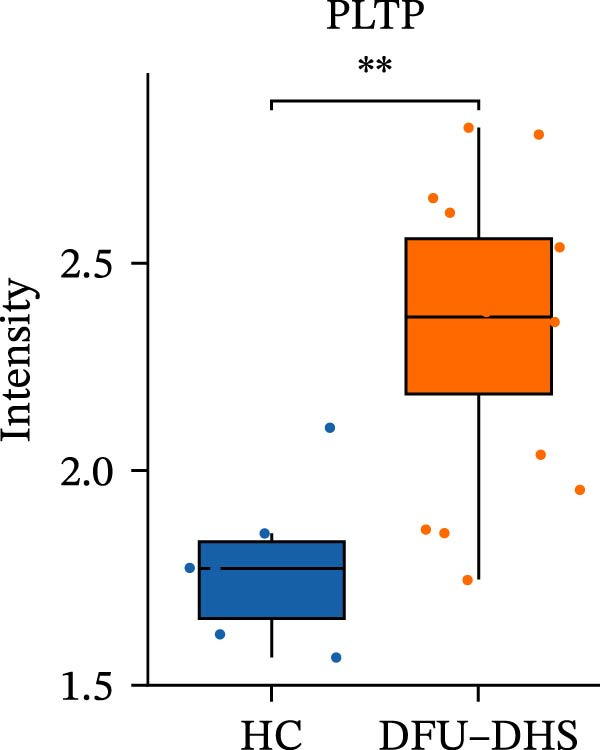
(E)

### 3.5. PPI Network Analysis of DEPs

**Figure 5:**
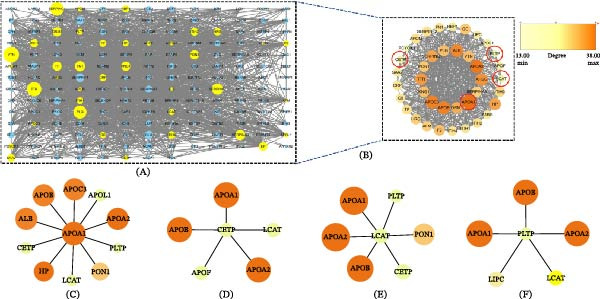
PPI network and module analysis. (A) String PPI network for DEPs. (B) The key cluster DEPs of PPI network, including APOA1, CETP, LCAT, and PLTP, which analyzed by MCODE in Cytoscape. (C) The key module containing APOA1 in the PPI network. (D) The key module containing CETP in the PPI network. (E) The key module containing LCAT in the PPI network. (F) The key module containing LCAT in the PPI network. Different colored nodes represent differentially expressed proteins.

We next constructed a PPI network to identify potential hub proteins and clusters within the DFU–DHS proteomic changes. Using the STRING database (confidence > 0.7) and Cytoscape, we generated a network comprising 193 nodes and 1777 edges (interactions) among the DEPs (Figure 5A). The network was then analyzed with the molecular complex detection (MCODE) algorithm. Subsequently, we have defined the critical module containing 39 nodes and 511 edges which scored 26.895. This top module contained four proteins: APOA1, LCAT, PLTP, and CETP. These dysregulated, HDL‐related proteins form a central cluster of tightly co‐expressed or interacting proteins in DFU–DHS. In the network shown in Figure 5C–F, APOA1, CETP, LCAT, and PLTP and other proteins also exhibit various interactions, highlighting the correlation and significance of these four proteins.

### 3.6. ELISAs Validate the Increase of APOA1, LCAT, PLTP, and CETP in DFU Patients With DHS Serum and Their Ability to Predict Disease Severity

In order to verify the results in a larger patient sample, we conducted a questionnaire survey among patients with DFUs (Table S2). Based on the results of the Table 3 questionnaire, we selected patients exhibiting symptoms meeting at least three of the following characteristics: heaviness in the head and body, upper abdominal distension, thirst without desire to drink, a swollen and soft tongue with a yellow greasy coating, and a slippery or rapid pulse, all rated as “Medium” or “Heavy” in severity. We validated the proteomic findings by measuring the serum levels of APOA1, LCAT, PLTP, and CETP in an independent cohort of 28 DFU–DHS patients using ELISA. Consistent with our discovery data, all four proteins were significantly dysregulated in DFU–DHS patients compared with HCs. ROC curve analysis demonstrated that these proteins possess good diagnostic power for distinguishing DFU–DHS patients, with individual AUC of 0.7964 for APOA1, 0.7321 for LCAT, 0.8875 for PLTP, and 0.8143 for CETP (Figure 5A–D). Notably, the combination of all four biomarkers achieved an AUC of 0.9679 and the results show a very large effect size (Youden’s index > 0.9), suggesting superior diagnostic performance (Figure 6E and Table 4).

Figure 6Elisa verification of DEPs and disease prediction ability. Elisa verification of APOA1 (A), LCAT (B), CETP (C), and PLTP (D). (E) ROC curve of APOA1, LCAT, CETP, PLTP, and combination of proteins.  ^∗^ *p* < 0.05;  ^∗∗^ *p* < 0.01;  ^∗∗∗^ *p* < 0.001.

**Figure:**
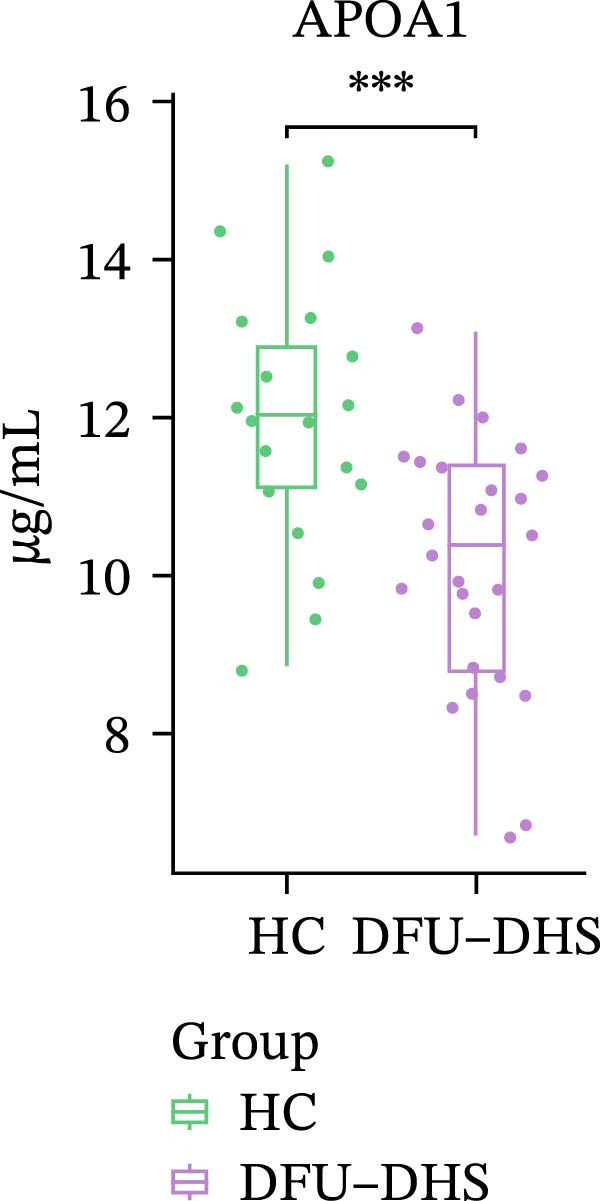
(A)

**Figure:**
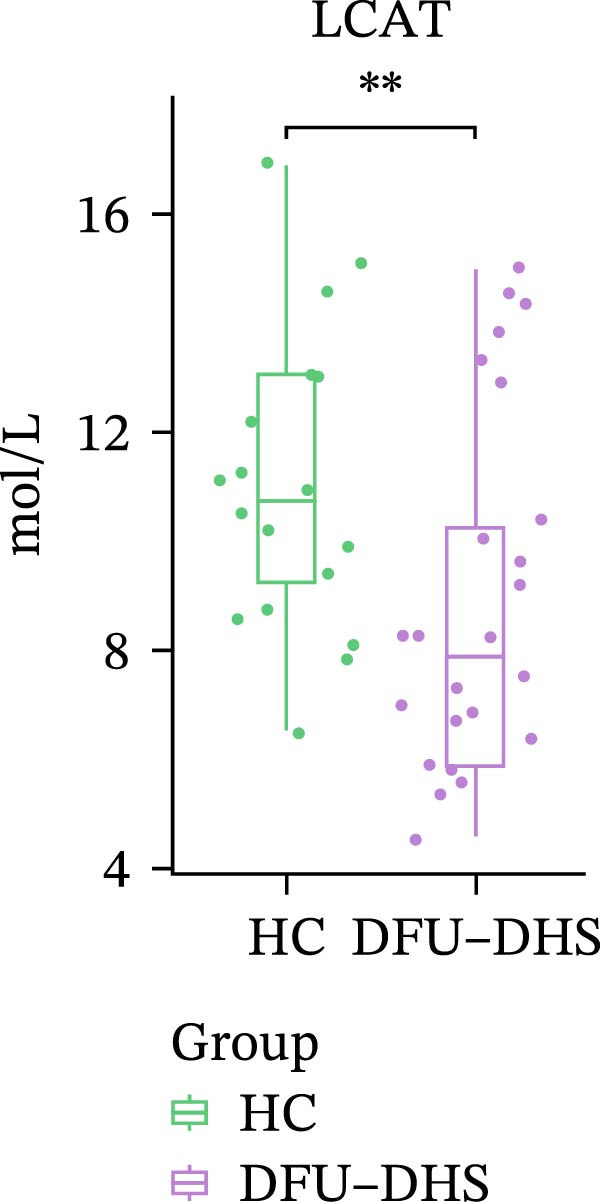
(B)

**Figure:**
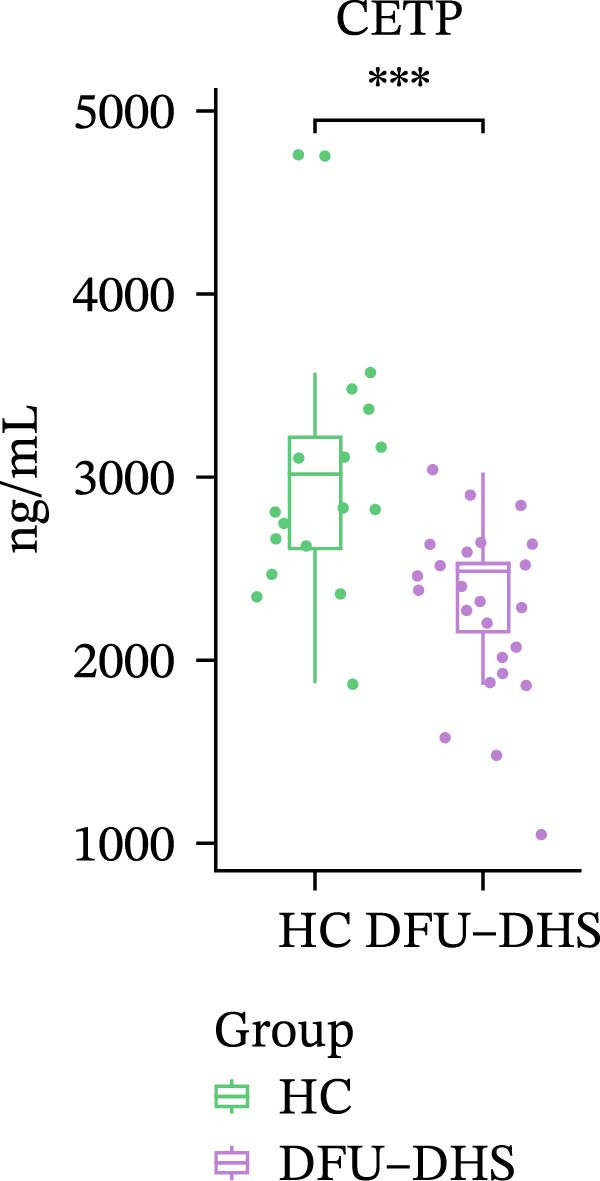
(C)

**Figure:**
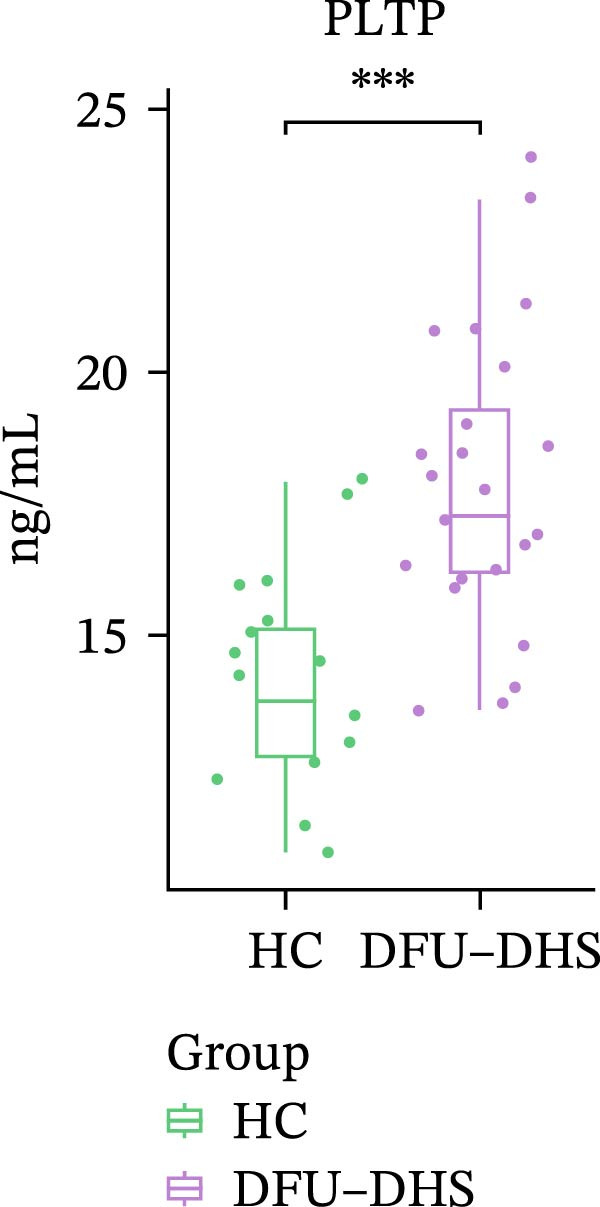
(D)

**Figure:**
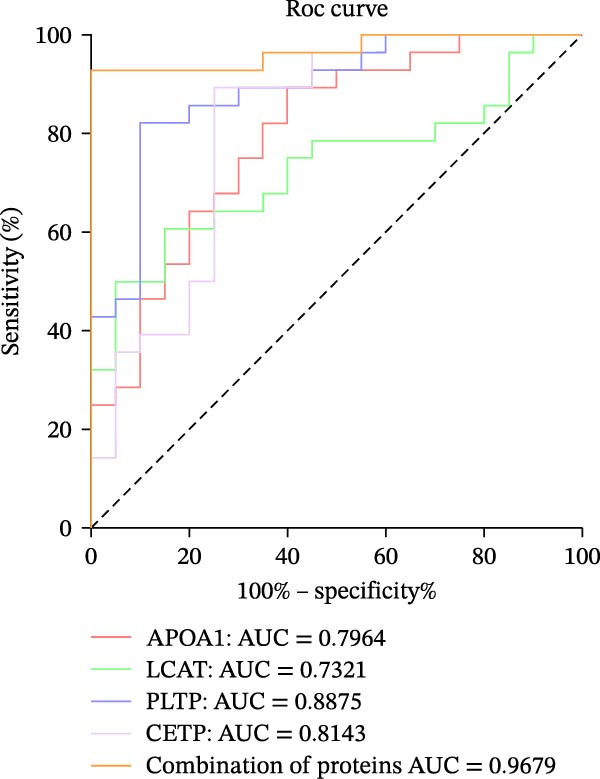
(E)

**Table 3:** Dampness–heat syndrome related characteristics in patients with diabetic foot ulcer.

Check indicators	Symptoms	Light (cases)	Medium (cases)	Heavy (cases)	Percentage (%)
General symptoms	Heaviness in head and drowsiness	13	4	2	70.37
	Sensory perception and heat sensation	5	4	1	37.04
	Tiredness and weakness	9	7	2	66.67
	Thirst and drinking more	12	6	2	74.07
	Abdominal distension	6	5	3	51.85
Local wound symptoms	Wound exudate	3	5	10	66.67
	Redness and swelling of the surrounding tissues around the wound	7	8	6	77.78
	Wound pain	6	12	7	92.59
	Granules and odor	11	7	2	74.07
Tongue and pulse	Tongue	3	22	0	92.59
	Pulse	8	15	1	88.89

**Table 4:** Diagnostic accuracy in the diagnosis of diabetic foot ulcer patients with dampness–heat syndrome using serum biomarkers.

Name	DFU–DHS	HC	Cut‐Off	Sensitivity (%)	Specificity (%)	AUC	Youden’s index
APOA1	10.24 ± 1.65	11.95 ± 1.62	11.76	89.29	60.00	0.7964	0.4929
LCAT	8.69 ± 3.28	11.05 ± 2.73	8.42	60.71	85.00	0.7321	0.4571
CETP	2327.17 ± 461.87	3039.86 ± 746.23	2654.00	89.29	75.00	0.8143	0.6429
PLTP	17.63 ± 2.89	13.94 ± 1.92	16.05	82.14	90.00	0.8875	0.7214
Combination of proteins	/	/	0.97	92.86	100.00	0.9679	0.9286

Abbreviations: APO1, apolipoprotein A1; CETP, cholesteryl ester transfer protein; LCAT, lecithin–cholesterol acyltransferase; PLTP, phospholipid transfer protein.

### 3.7. Correlations Between the Serum Levels of Target Proteins and Clinical Indexes in Diabetic Foot Patients With DHS

Finally, we investigated how the serum levels of the four validated proteins correlated with clinical indexes of disease severity and metabolism in the DFU–DHS patients. Figure 7 shows a correlation heatmap analyzing the Pearson correlation coefficients between APOA1, LCAT, PLTP, and CETP and various clinical measurements (including inflammatory markers and metabolic parameters). Overall, higher levels of the apolipoproteins tended to be associated with a more favorable clinical profile. Notably, APOA1 and LCAT concentrations were inversely correlated with markers of inflammation: for example, in DFU–DHS patients, those with higher APOA1 levels had lower erythrocyte sedimentation rates (ESRs), TGs and total cholesterol (*r* = −0.65, *p* = 0.001), indicating an anti‐inflammatory, lipid‐lowering effect. Although more moderate in strength, LCAT levels were similarly negative correlated with the ESR. In contrast, PLTP was positively correlated with measures of poor metabolic control. In other words, DFU–DHS patients with elevated PLTP tended to have worse glycemic control and hyperlipidemia. We observed significant positive correlations between LCAT and HDL‐C (*r* = 0.57, *p* < 0.001), as well as between CETP and HDL‐C levels (*r* = 0.56, *p* < 0.001). CETP also tended to be correlated with LDL‐C and VLDL‐C levels, which is consistent with its role in lipid exchange between lipoproteins. Collectively, these correlation results suggest that the identified biomarkers not only reflect the degree of inflammation and metabolic disorders within the patient group but also distinguish DFU–DHS patients from healthy individuals.

Figure 7Correlations between serum levels of target proteins and clinical indexes in diabetic foot ulcer patients with dampness–heat syndrome. (A) Correlation‐heatmap between serum levels of target proteins and clinical indexes in the DFU–DHS group. (B) Negative correlations between LCAT and TC in the DFU–DFS group. (C) Negative correlations between APOA1 and TC in the DFU–DHS group. (D) Positive correlations between CETP and HDL‐C in the DFU–DHS group. (E) Positive correlations between LCAT and HDL‐C in the DFU–DHS group.  ^∗^ *p* < 0.05;  ^∗∗^ *p* < 0.01;  ^∗∗∗^ *p* < 0.001.

**Figure:**
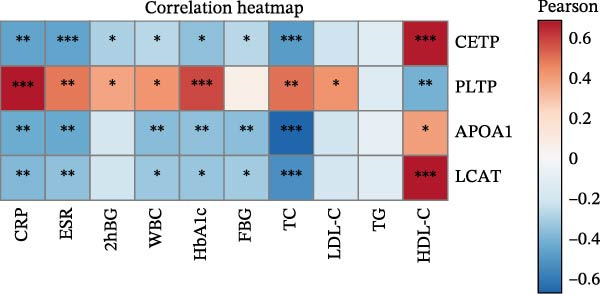
(A)

**Figure:**
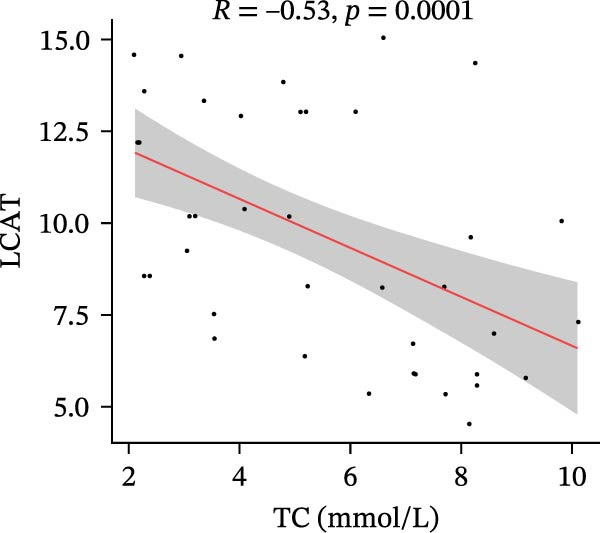
(B)

**Figure:**
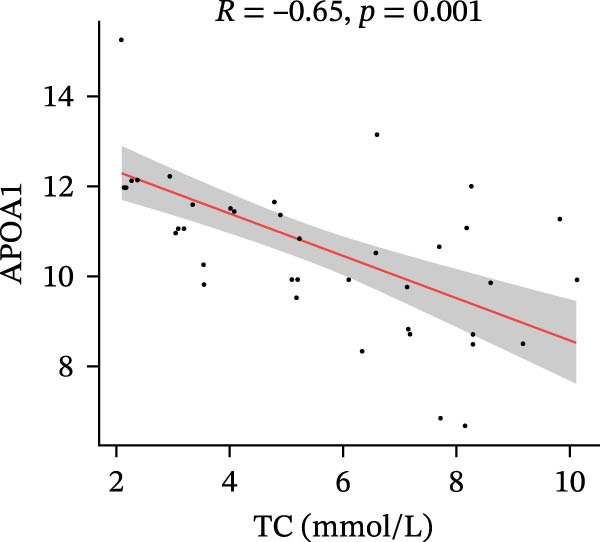
(C)

**Figure:**
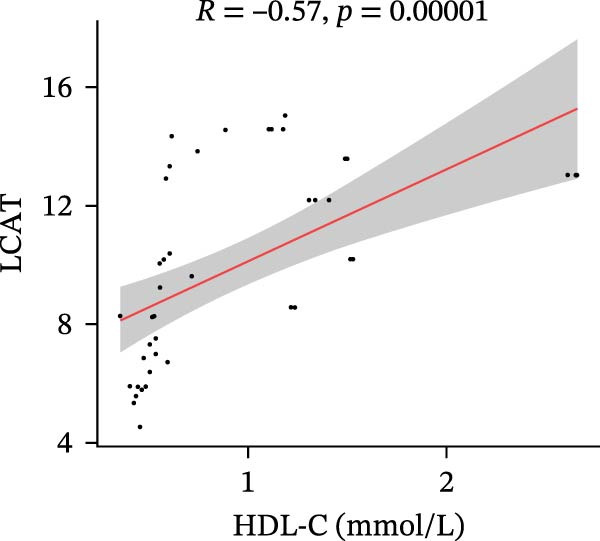
(D)

**Figure:**
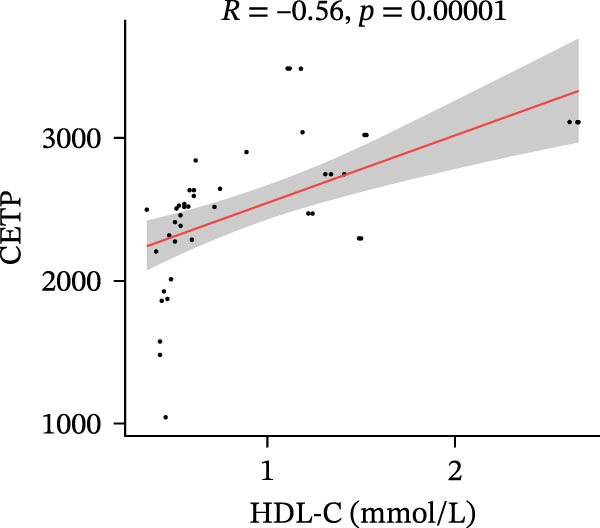
(E)

## 4. Discussion

### 4.1. Lipid Metabolic Dysregulation: The Molecular Cornerstone of DHS

Previous studies on DFU patients with DHS have linked disease pathology to markers of systemic oxidative stress and inflammation, including carbon‐modified proteins and total antioxidant capacity. While tissue biopsies directly assess local pathophysiology, they may inadequately capture the systemic metabolic dysregulation inherent to DHS, such as chronic low‐grade inflammation and lipid metabolism disorders [25–29]. In TCM theory, the pathogenesis of DHS is rooted in “spleen deficiency with dampness–heat,” which is thought to manifest systemically as insulin resistance and oxidative stress, and locally as inflammation (e.g., pedal erythema and edema) [30]. As a circulating medium, serum integrates disease‐relevant biomarkers, encompassing oxidative stress mediators, inflammatory cytokines, and metabolites [31]. Our study employed 4D‐DIA proteomics to delineate plasma protein profiles in DFU–DHS patients, identifying 201 DEPs versus HCs. GO enrichment analysis of the DEPs revealed significant involvement in HDL particle remodeling and reverse cholesterol transport, indicating profound abnormalities in lipid metabolism in DFU–DHS patients. TCM classifies dampness–heat diabetic foot syndrome as “Dampness–heat toxin syndrome,” with a core pathology involving “Dampness–heat accumulation and Qi‐blood stagnation,” frequently compounded by spleen deficiency, phlegm‐dampness, or liver/kidney yin deficiency. Contemporary research has confirmed that DHS correlates with chronic low‐grade inflammation, aberrant lipid metabolism (e.g., elevated free fatty acids), and oxidative stress, forming a pathogenic “dampness–heat–lipotoxicity–inflammation” cycle [32, 33]. Metabolomic studies of this research further demonstrated significant abnormalities in serum glycerophospholipids, sphingomyelins, and arachidonic acid derivatives in DHS patients, directly linking lipid dysmetabolism to TCM‐defined “dampness–heat” pathology. Tissue edema in DFU, which corresponds to the TCM concept of “dampness,” may partly arise from interstitial lipid deposition secondary to impaired lipoprotein lipase activity and reduced TG clearance. In turn, disorders of lipid metabolism downregulate PPAR*γ* activity, reducing vascular smooth muscle cell differentiation and affecting neovascularization [34]. KEGG pathway enrichment analysis indicates the main enrichment of DEPs are involved in complement and coagulation cascade reactions, as well as the cholesterol metabolism pathway, suggesting that patients with dampness–heat type diabetic foot have abnormal cholesterol metabolism and abnormal coagulation function. This finding also confirms from the perspective of overall systemic metabolism that lipid metabolism disorders are closely related to the pathology of diabetic foot patients with DHS.

### 4.2. Significance of the Identified Biomarkers in DFU With DHS

Timely diagnosis and precise subtype stratification of DFU are crucial for preventing disease progression and reducing amputation. However, traditional inflammatory indicators, such as CRP, procalcitonin, white blood cell count, interleukin‐6, and the ESR, primarily reflect the presence and severity of infection [35]. While these markers increase significantly in infected (wet gangrene) patients compared with noninfected (dry gangrene) DFU patients, they do not discriminate the underlying syndrome type (dampness–heat vs. other TCM subtypes) and are not specific to the metabolic/inflammatory milieu of DHS [36]. In fact, a dampness–heat constitution is relatively common in the diabetic population (~9% in one epidemiological survey), yet clinicians currently lack dedicated biomarkers to identify DFU–DHS or guide tailored therapies [37].

It is widely acknowledged that the effectiveness of a biomarker is contingent upon the delineation of the disease state. This study analyzed one of the main enriched KEGG pathways of DEPs in patients with DHS diabetic foot disease–lipid metabolism. The proteins of interest were APOA1, PLTP, LCAT, and CETP. After ELISA analysis of serum samples from 28 patients was conducted, it was confirmed that APOA1, PLTP, LCAT, and CETP had high sensitivity and specificity, and could be used as multiple potential biomarkers for dampness–heat type diabetic foot.

APOA1 is the major apolipoprotein of HDL and a crucial mediator of reverse cholesterol transport from peripheral tissues to the liver [38]. Elevated APOA1 has been linked to improved glycemic control in individuals with T2D and a reduced risk of progression from prediabetes [39]. In our cohort, APOA1 levels were inversely correlated with markers of inflammation and metabolism (higher APOA1 was associated with lower ESR, 2‐h postprandial blood glucose, and TGs), reinforcing the concept that APOA1 confers a protective, anti‐inflammatory effect under dampness/heat conditions. Moreover, low APOA1 is a known risk factor in various diseases (e.g., a potential indicator of aplastic anemia, metabolic side effects of schizophrenia, and certain cancers), partly due to its role in promoting cholesterol efflux and preventing foam cell formation [40]. Taken together, these findings suggest that APOA1 mitigates the “dampness–heat” pathological process through lipid regulation, anti‐inflammatory, and immunomodulatory functions [41]. The decreased levels of APOA1 in DFU–DHS patients likely contribute to heightened inflammation and dyslipidemia, positioning APOA1 not only as a valuable biomarker but also as a candidate for therapeutic interventions.

LCAT is the sole enzyme responsible for esterifying free cholesterol to cholesteryl esters in plasma, a reaction fundamental to lipoprotein homeostasis [42, 43]. LCAT activity directly influences the size and composition of HDL particles: deficiency leads to the accumulation of immature pre*β*‐HDL, while overexpression results in the formation of large HDL1 particles rich in APOE, significantly increasing plasma total cholesterol and HDL cholesterol levels [44]. Beyond lipid metabolism, LCAT exhibits concentration‐dependent antioxidant activity during the oxidation of VLDL, but this may come at the expense of HDL’s antioxidant properties [45, 46]. Oxidatively modified HDL can promote a pro‐inflammatory state, which pathologically aligns with conditions like diabetic “dampness–heat accumulation,” characterized by heightened oxidative stress and lipid toxicity [46, 47]. In DFU–DHS patients, an imbalance in LCAT could therefore contribute to the dysfunctional HDL and increased oxidative stress observed in this syndrome. Additionally, emerging evidence suggests LCAT may influence glucose and energy metabolism: for example, LCAT deficiency has been reported to promote the browning of adipose tissue in animal models, linking it to metabolic diseases such as obesity‐related insulin resistance and diabetic complications.

PLTP facilitates the transfer of phospholipids between lipoproteins and plays a role in HDL remodeling. In T2D and metabolic syndrome, elevated PLTP activity is correlated with insulin resistance, high TG levels, and central obesity (e.g., increased waist‐to‐hip ratio) [48]. It is considered an independent predictor of insulin sensitivity and cardiovascular risk. Clinically, PLTP activity has been used to distinguish diabetes subtypes and monitor therapeutic responses: for example, insulin therapy tends to decrease PLTP activity, but this effect is blunted in patients with significant insulin resistance, indicating that PLTP reflects the efficacy of metabolic control [49]. In our DFU–DHS patients, PLTP levels were positively correlated with HbA1c and TG levels. These findings suggest that elevated PLTP is associated with poorer glycemic control and hyperlipidemia, aligning with the TCM concept of DHS where “heat” stems from uncontrolled diabetes and “dampness” from lipid excess.

CETP primarily facilitates the exchange of cholesteryl esters and TGs between HDL and apoB‐containing lipoproteins (LDL and VLDL). In general, lower CETP activity leads to increased HDL‐C and reduced LDL‐C; interestingly, studies have shown that diabetic patients often exhibit reduced CETP activity, which might seem beneficial for lipid profiles but also indicates a disruption in normal cholesterol trafficking. Pharmacologically, CETP is a validated target such as anacetrapib (CETP inhibitor), which has been shown to significantly lower non‐HDL cholesterol and reduce cardiovascular events (as demonstrated in the REVEAL trial) [50]. These outcomes support the idea that modifying CETP activity can impact disease, yet they also highlight that the role of CETP is context dependent. As a biomarker, CETP must be interpreted with caution because its levels or activity can be influenced by factors like obesity and smoking. For example, smokers exhibit inconsistent changes in CETP activity, undermining its reliability as a standalone indicator. In our study, we observed a significant dysregulation of CETP in DFU–DHS patients (with ELISA suggesting an elevation in CETP levels compared with those in controls) [51]. This might reflect a compensatory response to the disturbed lipid environment; despite the lower functional activity reported in diabetic patients, the increased protein level could be due to feedback mechanisms or inflammatory stimuli [52]. Although CETP may be the least specific of the four markers for DHS, its inclusion in the panel adds considerable value—likely by capturing aspects of abnormal lipid exchange and transport not fully represented by APOA1, LCAT, and PLTP.

## 5. Limitations of the Study

Our study has certain limitations. First, the number of plasma samples was modest, and the findings should be validated in larger, independent cohorts. Second, although the control group was matched to the DFU–DHS cohort, a more informative design would compare patients with diabetic foot without DHS as well as those with other syndrome types. Third, potential confounders and sources of bias were not fully characterized and should be explicitly addressed in future analyses. Finally, cellular‐level validation has not yet been completed, and those experiments are ongoing.

## 6. Conclusion

DFU with DHS is a prevalent condition characterized by local redness, swelling, heat, pain, exudative ulcers, a high risk of infection, and systemic metabolic dysregulation. This study represents the first application of 4D‐DIA proteomics to investigate the serum proteomic profile of DFU patients with DHS. Our analysis identified 201 DEPs between DHS patients and HCs. Bioinformatics enrichment analysis consistently pointed to disruptions in lipid metabolism, particularly in HDL remodeling and reverse cholesterol transport, as well as in complement and coagulation cascades. We focused on and validated a panel of four apolipoproteins: APOA1, LCAT, PLTP, and CETP, which are central to cholesterol metabolism. The combined diagnostic power of these biomarkers yielded an impressive AUC, suggesting their strong potential for the objective stratification of DHS in DFU patients.

**Nomenclature**
4D‐DIA: Four‐dimensional data‐independent acquisition
APOA1: Apolipoprotein A‐1
AUC: Area under the curve
CETP: Cholesteryl ester transfer protein
CRP: C‐reactive protein
DEPs: Differentially expressed proteins
DFU: Diabetic foot ulcers
DHS: Dampness–heat syndrome
FC: Fold change
FDR: False discovery rate
FGL1: Fibrinogen like 1
GO: Gene Ontology
HC: Healthy control
HDL: High‐density lipoprotein
IDL: Intermediate density lipoprotein
LC–MS/MS: Liquid chromatography–tandem mass spectrometry
LDL: Low‐density lipoprotein
LCAT: Lecithin–cholesterol acyltransferase
PLTP: Phospholipid transfer protein
ROC: Receiver operating characteristic
SAA1: Serum amyloid A1
SAA2: Serum amyloid A2
S100A8: S100 calcium binding protein A8
T2D: Type 2 diabetes
TCM: Traditional Chinese medicine
VLDL: Very low‐density lipoprotein.

## Author Contributions

Investigation: Jiawei Feng. Data curation: Shiyu Wang. Writing – original draft preparation: Jinlun Jiang. Writing – review and editing: Cheng Zhao. Visualization: Mingmei Zhou. Study conception and design: Yiming Ni.

## Funding

This research was funded by the Shanghai Science and Technology Commission, National Natural Science Foundation of Shanghai (Grant 23ZR1460300), the Shanghai Municipal Science and Technology Commission, Medical Innovation Research Project (Grant 22Y11922700), the Shanghai Municipal Health Commission, General Program (Grant 202240386), the Shanghai Science and Technology Development Fund, Science and Technology Innovation Project (Grant AXZ‐1), the Shanghai Municipal Science and Technology Commission, Science and Technology Support Project of Biomedicine (Grant 21S21900100), the Shanghai Municipal Health Commission, Key Construction Project of TCM Disease (Diabetic Foot) (Grant 2025ZDBZ26), Hongkou District Health Commission of Shanghai (Grants HKGYQYXM‐2026‐70 and HKGYQYXM‐2026‐71).

## Disclosure

All the authors have read and agreed to the published version of the manuscript. All aspects of the work, including data collection, analysis, writing, and editing, were performed solely by the authors.

## Conflicts of Interest

All authors declare no conflicts of interest.

## Supporting Information

Additional supporting information can be found online in the Supporting Information section.

## Supporting information


**Supporting Information** Supporting Information Table S1. List of proteins with statistically significant changes in abundance in the DFU–DHS of patients compared to HC. This table lists all the differentially expressed proteins, including 53 upregulated proteins and 148 downregulated proteins. Table S2. TCM syndromes information questionnaire for diabetes foot ulcers with dampness–heat syndrome patients. This table is designed to collect information from patients, and then select appropriate patient samples for verification.

## Data Availability

The data that support the findings of this study are available from the corresponding author upon reasonable request.
