# Supplementary material for: Analysis of Biomarkers in Diabetic Foot Ulcer Patients With Dampness–Heat Syndrome Based on 4D‐DIA Proteomics Technology
Source: J Diabetes Res. 2026 Mar 25;2026:6604989. doi: 10.1155/jdr/6604989 (PMC13140316; doi:10.1155/jdr/6604989)
Supplement: Supplementary file 1 — Supporting Information Supporting Information Table S1. List of proteins with statistically significant changes in abundance in the DFU–DHS of patients compared to HC. This table lists all the differentially expressed proteins, including 53 upregulated proteins and 148 downregulated proteins. Table S2. TCM syndromes information questionnaire for diabetes foot ulcers with dampness–heat syndrome patients. This table is designed to collect information from patients, and then select appropriate patient samples for verification. [file JDR-2026-6604989-s001.docx]

**Table 1.** List of proteins with statistically significant changes in abundance in the DFU-DHS of patients compared to HC.

| **Protein** | **Gene Name** | **FC** | **log2FC** | ***p-***  ***value*** |
| --- | --- | --- | --- | --- |
| Serum amyloid A-2 protein | *SAA2* | 27.058 | 4.758 | 0.002 |
| Serum amyloid A-1 protein | *SAA1* | 13.767 | 3.783 | 0.003 |
| C-reactive protein | *CRP* | 8.984 | 3.167 | 0.001 |
| Fibrinogen-like protein 1 | *FGL1* | 5.026 | 2.330 | 0.001 |
| Protein S100-A8 | *S100A8* | 4.232 | 2.081 | 0.001 |
| Insulin-like growth factor-binding protein 2 | *IGFBP2* | 3.501 | 1.808 | 0.000 |
| Protein S100-A9 | *S100A9* | 3.000 | 1.585 | 0.007 |
| Apolipoprotein F | *APOF* | 2.893 | 1.533 | 0.000 |
| Myeloblastin | *PRTN3* | 2.804 | 1.488 | 0.038 |
| Procollagen C-endopeptidase enhancer 1 | *PCOLCE* | 2.647 | 1.404 | 0.000 |
| Serine/threonine-protein kinase/endoribonuclease IRE1 | *ERN1* | 2.577 | 1.366 | 0.005 |
| Chromogranin-A | *CHGA* | 2.416 | 1.272 | 0.008 |
| Insulin-like growth factor-binding protein 1 | *IGFBP1* | 2.139 | 1.097 | 0.019 |
| Resistin | *RETN* | 2.072 | 1.051 | 0.027 |
| Putative sodium-coupled neutral amino acid transporter 10 | *SLC38A10* | 1.996 | 0.997 | 0.019 |
| Nucleobindin-1 | *NUCB1* | 1.980 | 0.985 | 0.002 |
| Multimerin-1 | *MMRN1* | 1.976 | 0.983 | 0.045 |
| Haptoglobin | *HP* | 1.936 | 0.953 | 0.000 |
| Asialoglycoprotein receptor 2 | *ASGR2* | 1.928 | 0.947 | 0.003 |
| Progranulin | *GRN* | 1.917 | 0.939 | 0.001 |
| Immunoglobulin heavy variable 3-20 | *IGHV3-20* | 1.893 | 0.920 | 0.027 |
| Probable non-functional immunoglobulin heavy variable 3-38 | *IGHV3-38* | 1.878 | 0.909 | 0.019 |
| Cadherin-2 | *CDH2* | 1.841 | 0.880 | 0.000 |
| Immunoglobulin heavy variable 3-43 | *IGHV3-43* | 1.804 | 0.851 | 0.022 |
| Complement factor H-related protein 4 | *CFHR4* | 1.799 | 0.847 | 0.043 |
| Immunoglobulin lambda constant 7 | *IGLC7* | 1.788 | 0.838 | 0.029 |
| Out at first protein homolog | *OAF* | 1.766 | 0.820 | 0.001 |
| Insulin-like growth factor-binding protein 4 | *IGFBP4* | 1.743 | 0.802 | 0.017 |
| Integral membrane protein 2B | *ITM2B* | 1.716 | 0.779 | 0.018 |
| Alpha-1-antichymotrypsin | *SERPINA3* | 1.692 | 0.759 | 0.019 |
| Neuronal cell adhesion molecule | *NRCAM* | 1.673 | 0.743 | 0.000 |
| Cadherin-1 | *CDH1* | 1.669 | 0.739 | 0.005 |
| Leucine-rich alpha-2-glycoprotein | *LRG1* | 1.660 | 0.732 | 0.006 |
| Laminin subunit gamma-1 | *LAMC1* | 1.641 | 0.715 | 0.025 |
| Beta-2-microglobulin | *B2M* | 1.594 | 0.672 | 0.011 |
| CD59 glycoprotein | *CD59* | 1.590 | 0.669 | 0.026 |
| Dystroglycan 1 | *DAG1* | 1.586 | 0.666 | 0.014 |
| Mimecan | *OGN* | 1.573 | 0.653 | 0.031 |
| ADAM DEC1 | *ADAMDEC1* | 1.533 | 0.616 | 0.017 |
| EGF-containing fibulin-like extracellular matrix protein 2 | *EFEMP2* | 1.523 | 0.607 | 0.012 |
| Desmocollin-2 | *DSC2* | 1.505 | 0.589 | 0.006 |
| Arginase-1 | *ARG1* | 1.483 | 0.569 | 0.035 |
| Oncoprotein-induced transcript 3 protein | *OIT3* | 1.474 | 0.560 | 0.001 |
| Immunoglobulin kappa variable 3-20 | *IGKV3-20* | 1.431 | 0.517 | 0.036 |
| Inter-alpha-trypsin inhibitor heavy chain H3 | *ITIH3* | 1.394 | 0.479 | 0.042 |
| Hypoxia up-regulated protein 1 | *HYOU1* | 1.380 | 0.465 | 0.044 |
| Cathepsin D | *CTSD* | 1.373 | 0.457 | 0.027 |
| Complement component C9 | *C9* | 1.346 | 0.429 | 0.037 |
| Phospholipid transfer protein | *PLTP* | 1.338 | 0.420 | 0.001 |
| Tenascin | *TNC* | 1.334 | 0.415 | 0.021 |
| Alpha-1-acid glycoprotein 1 | *ORM1* | 1.332 | 0.414 | 0.036 |
| Laminin subunit beta-1 | *LAMB1* | 1.330 | 0.412 | 0.029 |
| Neutral alpha-glucosidase AB | *GANAB* | 1.298 | 0.376 | 0.019 |
| Endoplasmic reticulum chaperone BiP | *HSPA5* | 0.872 | -0.197 | 0.028 |
| Complement C1r subcomponent | *C1R* | 0.815 | -0.294 | 0.008 |
| Lysosome-associated membrane glycoprotein 2 | *LAMP2* | 0.812 | -0.300 | 0.037 |
| Alpha-1B-glycoprotein | *A1BG* | 0.811 | -0.303 | 0.019 |
| Complement C1s subcomponent | *C1S* | 0.806 | -0.312 | 0.003 |
| Complement component C6 | *C6* | 0.805 | -0.313 | 0.041 |
| Plasma alpha-L-fucosidase | *FUCA2* | 0.804 | -0.315 | 0.048 |
| Hyaluronan-binding protein 2 | *HABP2* | 0.801 | -0.319 | 0.035 |
| Complement component C8 beta chain | *C8B* | 0.792 | -0.337 | 0.047 |
| Coagulation factor X | *F10* | 0.786 | -0.347 | 0.024 |
| Pyruvate kinase PKM | *PKM* | 0.765 | -0.386 | 0.034 |
| Multiple inositol polyphosphate phosphatase 1 | *MINPP1* | 0.763 | -0.390 | 0.004 |
| Neural cell adhesion molecule 1 | *NCAM1* | 0.762 | -0.392 | 0.035 |
| Corticosteroid-binding globulin | *SERPINA6* | 0.758 | -0.399 | 0.004 |
| Neural cell adhesion molecule 2 | *NCAM2* | 0.758 | -0.400 | 0.040 |
| Kininogen-1 | *KNG1* | 0.758 | -0.400 | 0.002 |
| Complement component C8 gamma chain | *C8G* | 0.756 | -0.403 | 0.009 |
| Endoglin | *ENG* | 0.756 | -0.404 | 0.021 |
| Integrin beta-1 | *ITGB1* | 0.749 | -0.417 | 0.041 |
| Vitamin K-dependent protein C | *PROC* | 0.748 | -0.419 | 0.015 |
| Lumican | *LUM* | 0.747 | -0.422 | 0.042 |
| Lysosome-associated membrane glycoprotein 1 | *LAMP1* | 0.743 | -0.428 | 0.031 |
| Complement component C8 alpha chain | *C8A* | 0.743 | -0.429 | 0.011 |
| Protein phosphatase 1 regulatory subunit 3A | *PPP1R3A* | 0.741 | -0.433 | 0.002 |
| Clusterin | *CLU* | 0.739 | -0.436 | 0.001 |
| N-acetyllactosaminide beta-1,3-N-acetylglucosaminyltransferase 2 | *B3GNT2* | 0.736 | -0.443 | 0.010 |
| Vitamin D-binding protein | *GC* | 0.732 | -0.451 | 0.000 |
| Coagulation factor VII | *F7* | 0.731 | -0.452 | 0.034 |
| Vitronectin | *VTN* | 0.725 | -0.464 | 0.001 |
| Inhibin beta C chain | *INHBC* | 0.724 | -0.466 | 0.025 |
| Epidermal growth factor receptor | *EGFR* | 0.723 | -0.467 | 0.022 |
| Immunoglobulin lambda-1 light chain | *IGLL1* | 0.716 | -0.483 | 0.001 |
| Inter-alpha-trypsin inhibitor heavy chain H4 | *ITIH4* | 0.714 | -0.486 | 0.008 |
| Glutathione peroxidase 3 | *GPX3* | 0.710 | -0.494 | 0.021 |
| Moesin | *MSN* | 0.702 | -0.509 | 0.022 |
| Apolipoprotein C-III | *APOC3* | 0.701 | -0.512 | 0.043 |
| Antithrombin-III | *SERPINC1* | 0.701 | -0.512 | 0.000 |
| Attractin | *ATRN* | 0.698 | -0.519 | 0.002 |
| Albumin | *ALB* | 0.695 | -0.525 | 0.028 |
| Intercellular adhesion molecule 3 | *ICAM3* | 0.694 | -0.527 | 0.015 |
| Ficolin-3 | *FCN3* | 0.685 | -0.545 | 0.046 |
| Dipeptidyl peptidase 4 | *DPP4* | 0.685 | -0.547 | 0.024 |
| Apolipoprotein D | *APOD* | 0.672 | -0.573 | 0.045 |
| Alpha-2-antiplasmin | *SERPINF2* | 0.671 | -0.575 | 0.000 |
| Dermokine | *DMKN* | 0.670 | -0.578 | 0.025 |
| Interleukin-1 receptor accessory protein | *IL1RAP* | 0.669 | -0.580 | 0.020 |
| Prothrombin | *F2* | 0.667 | -0.583 | 0.001 |
| Phosphatidylcholine-sterol acyltransferase | *LCAT* | 0.666 | -0.587 | 0.002 |
| Cathepsin F | *CTSF* | 0.664 | -0.591 | 0.045 |
| Di-N-acetylchitobiase | *CTBS* | 0.659 | -0.601 | 0.015 |
| Apolipoprotein L1 | *APOL1* | 0.659 | -0.603 | 0.011 |
| Fetuin-B | *FETUB* | 0.651 | -0.619 | 0.024 |
| Sulfhydryl oxidase 1 | *QSOX1* | 0.640 | -0.644 | 0.035 |
| Biotinidase | *BTD* | 0.639 | -0.645 | 0.001 |
| Carboxypeptidase Q | *CPQ* | 0.639 | -0.646 | 0.038 |
| ICOS ligand | *ICOSLG* | 0.638 | -0.648 | 0.007 |
| Plasma kallikrein | *KLKB1* | 0.638 | -0.649 | 0.008 |
| Apolipoprotein A-I | *APOA1* | 0.638 | -0.649 | 0.000 |
| Inter-alpha-trypsin inhibitor heavy chain H1 | *ITIH1* | 0.634 | -0.657 | 0.002 |
| Apolipoprotein B-100 | *APOB* | 0.630 | -0.666 | 0.030 |
| Exostosin-like 2 | *EXTL2* | 0.625 | -0.678 | 0.031 |
| Hepatocyte growth factor-like protein | *MST1* | 0.624 | -0.681 | 0.014 |
| Hepatic triacylglycerol lipase | *LIPC* | 0.622 | -0.685 | 0.006 |
| A disintegrin and metalloproteinase with thrombospondin motifs 13 | *ADAMTS13* | 0.620 | -0.690 | 0.006 |
| Tenascin-X | *TNXB* | 0.619 | -0.692 | 0.001 |
| Thyroxine-binding globulin | *SERPINA7* | 0.615 | -0.702 | 0.001 |
| Peroxiredoxin-6 | *PRDX6* | 0.614 | -0.705 | 0.012 |
| Anthrax toxin receptor 1 | *ANTXR1* | 0.608 | -0.718 | 0.015 |
| Inter-alpha-trypsin inhibitor heavy chain H2 | *ITIH2* | 0.605 | -0.725 | 0.001 |
| RNA-binding protein 25 | *RBM25* | 0.598 | -0.742 | 0.001 |
| Keratin, type II cytoskeletal 72;Keratin, type II cytoskeletal 71;Keratin, type II cytoskeletal 74;Keratin, type II cytoskeletal 73 | *KRT72* | 0.598 | -0.743 | 0.029 |
| Selenoprotein P | *SELENOP* | 0.594 | -0.751 | 0.002 |
| Pigment epithelium-derived factor | *SERPINF1* | 0.594 | -0.752 | 0.029 |
| Transthyretin | *TTR* | 0.592 | -0.757 | 0.005 |
| PDZ and LIM domain protein 1 | *PDLIM1* | 0.588 | -0.765 | 0.010 |
| Complement factor D | *CFD* | 0.588 | -0.767 | 0.020 |
| Serum paraoxonase/arylesterase 1 | *PON1* | 0.586 | -0.770 | 0.001 |
| Immunoglobulin kappa variable 2-28;Immunoglobulin kappa variable 2D-28 | *IGKV2-28* | 0.580 | -0.787 | 0.027 |
| L-lactate dehydrogenase B chain | *LDHB* | 0.579 | -0.788 | 0.002 |
| Programmed cell death 1 ligand 2 | *PDCD1LG2* | 0.576 | -0.796 | 0.002 |
| Contactin-3 | *CNTN3* | 0.576 | -0.796 | 0.007 |
| Fermitin family homolog 3 | *FERMT3* | 0.574 | -0.800 | 0.035 |
| Protein S100-A4 | *S100A4* | 0.574 | -0.801 | 0.003 |
| Cartilage acidic protein 1 | *CRTAC1* | 0.574 | -0.802 | 0.010 |
| Properdin | *CFP* | 0.572 | -0.806 | 0.023 |
| Mannan-binding lectin serine protease 1 | *MASP1* | 0.570 | -0.810 | 0.000 |
| Reticulon-4 receptor-like 2 | *RTN4RL2* | 0.567 | -0.818 | 0.004 |
| Fibroblast growth factor-binding protein 2 | *FGFBP2* | 0.566 | -0.821 | 0.022 |
| Complement factor H | *CFH* | 0.565 | -0.823 | 0.033 |
| Coagulation factor XII | *F12* | 0.564 | -0.826 | 0.019 |
| Transforming growth factor-beta-induced protein ig-h3 | *TGFBI* | 0.564 | -0.827 | 0.000 |
| Plasminogen | *PLG* | 0.561 | -0.834 | 0.004 |
| Retinol-binding protein 4 | *RBP4* | 0.558 | -0.842 | 0.011 |
| Heat shock 70 kDa protein 1A;Heat shock 70 kDa protein 1B | *HSPA1A* | 0.552 | -0.856 | 0.003 |
| Zyxin | *ZYX* | 0.552 | -0.858 | 0.033 |
| Serotransferrin | *TF* | 0.550 | -0.863 | 0.000 |
| Keratin, type II cytoskeletal 6B | *KRT6B* | 0.548 | -0.868 | 0.016 |
| Prolyl endopeptidase FAP | *FAP* | 0.547 | -0.870 | 0.004 |
| Tubulin beta-1 chain | *TUBB1* | 0.546 | -0.873 | 0.049 |
| Fibronectin | *FN1* | 0.545 | -0.876 | 0.007 |
| Apolipoprotein M | *APOM* | 0.540 | -0.888 | 0.004 |
| Creatine kinase M-type | *CKM* | 0.540 | -0.889 | 0.008 |
| Eukaryotic initiation factor 4A-I | *EIF4A1* | 0.536 | -0.899 | 0.035 |
| Mast/stem cell growth factor receptor Kit | *KIT* | 0.534 | -0.904 | 0.000 |
| Phosphatidylinositol-glycan-specific phospholipase D | *GPLD1* | 0.527 | -0.925 | 0.021 |
| Uromodulin | *UMOD* | 0.525 | -0.930 | 0.001 |
| Immunoglobulin kappa variable 1-27 | *IGKV1-27* | 0.522 | -0.938 | 0.033 |
| L-selectin | *SELL* | 0.511 | -0.969 | 0.000 |
| Cholinesterase | *BCHE* | 0.510 | -0.971 | 0.000 |
| BPI fold-containing family B member 1 | *BPIFB1* | 0.506 | -0.982 | 0.014 |
| Golgi-associated plant pathogenesis-related protein 1 | *GLIPR2* | 0.502 | -0.996 | 0.000 |
| Immunoglobulin lambda variable 3-16 | *IGLV3-16* | 0.499 | -1.003 | 0.001 |
| Endoplasmic reticulum aminopeptidase 2 | *ERAP2* | 0.499 | -1.004 | 0.000 |
| Cysteine-rich secretory protein 3 | *CRISP3* | 0.495 | -1.014 | 0.000 |
| Hepatocyte growth factor activator | *HGFAC* | 0.495 | -1.016 | 0.000 |
| Apolipoprotein A-II | *APOA2* | 0.494 | -1.017 | 0.000 |
| Gelsolin | *GSN* | 0.492 | -1.022 | 0.001 |
| Rab GDP dissociation inhibitor alpha | *GDI1* | 0.492 | -1.022 | 0.014 |
| Serine/threonine-protein phosphatase 2A activator | *PTPA* | 0.490 | -1.030 | 0.000 |
| Xaa-Pro dipeptidase | *PEPD* | 0.488 | -1.035 | 0.000 |
| Hematopoietic progenitor cell antigen CD34 | *CD34* | 0.483 | -1.051 | 0.006 |
| N-acetylmuramoyl-L-alanine amidase | *PGLYRP2* | 0.477 | -1.067 | 0.000 |
| Anthrax toxin receptor 2 | *ANTXR2* | 0.476 | -1.070 | 0.002 |
| Apolipoprotein E | *APOE* | 0.476 | -1.070 | 0.002 |
| Extracellular matrix protein 1 | *ECM1* | 0.468 | -1.095 | 0.000 |
| Noelin-2 | *OLFM2* | 0.466 | -1.101 | 0.001 |
| Tetranectin | *CLEC3B* | 0.466 | -1.102 | 0.000 |
| Immunoglobulin heavy chain | *IGHM* | 0.461 | -1.116 | 0.039 |
| Alpha-2-HS-glycoprotein | *AHSG* | 0.452 | -1.146 | 0.000 |
| Myosin light polypeptide 6 | *MYL6* | 0.434 | -1.203 | 0.001 |
| Adenylyl cyclase-associated protein 1 | *CAP1* | 0.430 | -1.219 | 0.007 |
| Apolipoprotein C-I | *APOC1* | 0.425 | -1.233 | 0.003 |
| Histidine-rich glycoprotein | *HRG* | 0.412 | -1.278 | 0.001 |
| Carboxypeptidase B2 | *CPB2* | 0.412 | -1.278 | 0.001 |
| Beta-Ala-His dipeptidase | *CNDP1* | 0.412 | -1.280 | 0.000 |
| Platelet glycoprotein Ib beta chain | *GP1BB* | 0.405 | -1.302 | 0.020 |
| Fructose-bisphosphate aldolase B | *ALDOB* | 0.405 | -1.305 | 0.006 |
| Afamin | *AFM* | 0.395 | -1.340 | 0.000 |
| Bone morphogenetic protein 1 | *BMP1* | 0.381 | -1.393 | 0.001 |
| Cholesteryl ester transfer protein | *CETP* | 0.380 | -1.397 | 0.000 |
| Prenylcysteine oxidase 1 | *PCYOX1* | 0.377 | -1.408 | 0.000 |
| Serum paraoxonase/lactonase 3 | *PON3* | 0.374 | -1.417 | 0.000 |
| Insulin-like growth factor-binding protein 5 | *IGFBP5* | 0.339 | -1.560 | 0.001 |
| Insulin-like growth factor-binding protein 3 | *IGFBP3* | 0.329 | -1.604 | 0.001 |
| Plasma serine protease inhibitor | *SERPINA5* | 0.308 | -1.699 | 0.002 |
| Kallistatin | *SERPINA4* | 0.300 | -1.739 | 0.000 |
| Insulin-like growth factor-binding protein complex acid labile subunit | *IGFALS* | 0.286 | -1.804 | 0.001 |
| Complement C1q tumor necrosis factor-related protein 3 | *C1QTNF3* | 0.277 | -1.852 | 0.000 |
| Pleckstrin | *PLEK* | 0.269 | -1.893 | 0.001 |
| Multivesicular body subunit 12B | *MVB12B* | 0.241 | -2.055 | 0.016 |

A fold change (FC) greater than 1.2 means a higher abundance in the diabetic foot of patients, while an FC less than 0.8 means lower abundance (*p* < 0.05) .

**Table 2.** TCM syndromes questionnaire for diabetes foot uclers with dampness-heat syndrome patients.

Please check the box that best fits your situation according to your actual situation in the last three months with“√”.

|  | **Symptoms** | **YES(level)** | | | **No** |
| --- | --- | --- | --- | --- | --- |
|  |  | **Light** | **Medium** | **Heavy** |  |
| General symptoms | Heaviness in head and drowsiness | Mild | Moderate | Severe |  |
|  | Sensory perception and heat sensation | Felt feverish, though temperature was normal. | Low fever (37.3℃ - 38℃) | Moderate fever (38.1℃ - 39℃) |  |
|  | Tiredness and weakness | Occasionally or Mild | Sometimes or Moderate | Frequently or  Sereve |  |
|  | Thirst and drinking more | Occasionally or Mild | Sometimes or Moderate | Frequently or  Sereve |  |
|  | Abdominal distension | Occasionally or Mild | Sometimes or Moderate | Frequently or  Sereve |  |
| Local wound symptoms | Wound exudate | Mild exudation | Moderate exudation | Severe exudation (pus exudation) |  |
|  | Redness and swelling of The surrounding tissues around the wound | Slight redness and swelling, with the area less than 2 cm in size | Clearly swollen, with a diameter of 2 to 5 cm, and increased skin temperature | Severe redness and swelling, with a diameter greater than 5 cm, spreading to the dorsum of the foot or the ankle, and significantly elevated skin temperature |  |
|  | Wound pain | Mild pain | Moderate pain | Intense pain |  |
|  | Granules and Odor | Dull and swollen | Grayish-white, dirty, with a slight odor | Necrosis, accompanied by a distinct pungent or rotten smell |  |
| Tongue and pulse | Tongue | Tongue is slightly red, and the coating is slightly yellow and greasy | Tongue is red, and the coating is yellow and thick and greasy | Tongue is red and purplish, with a yellow, greasy coating that is dry or scorched and yellowish |  |
|  | Pulse | Smooth or rapid pulse | Smooth and rapid pulse | Rapid and soggy or rapid and flooding |  |
